# Supplementary material for: A transferable machine-learning framework linking interstice distribution and plastic heterogeneity in metallic glasses
Source: Nat Commun. 2019 Dec 5;10:5537. doi: 10.1038/s41467-019-13511-9 (PMC6895099; doi:10.1038/s41467-019-13511-9)
Supplement: Supplementary file 1 — Supplementary Information [file 41467_2019_13511_MOESM1_ESM.pdf]

Supplementary Information

A transferable machine-learning framework linking interstice distribution and plastic heterogeneity in metallic glasses

Wang et al.

Outline

Supplementary Note 1. Details of the ML framework ... 3

Supplementary Note 2. Recursive feature elimination in Cu-Zr MGs ... 7

Supplementary Note 3. Symmetry functions and other existing features ... 9

Supplementary Note 4. Calibration curves of GBDT and Linear SVC models ... 13

Supplementary Note 5. ML results of Ni-Nb/Al-Sm/Fe-P MGs ... 15

Supplementary Note 6. Generalization results of ML models ... 16

Supplementary Note 7. Interpreting the feature sets ... 19

Supplementary References ... 37

Supplementary Note 1. Details of the ML framework

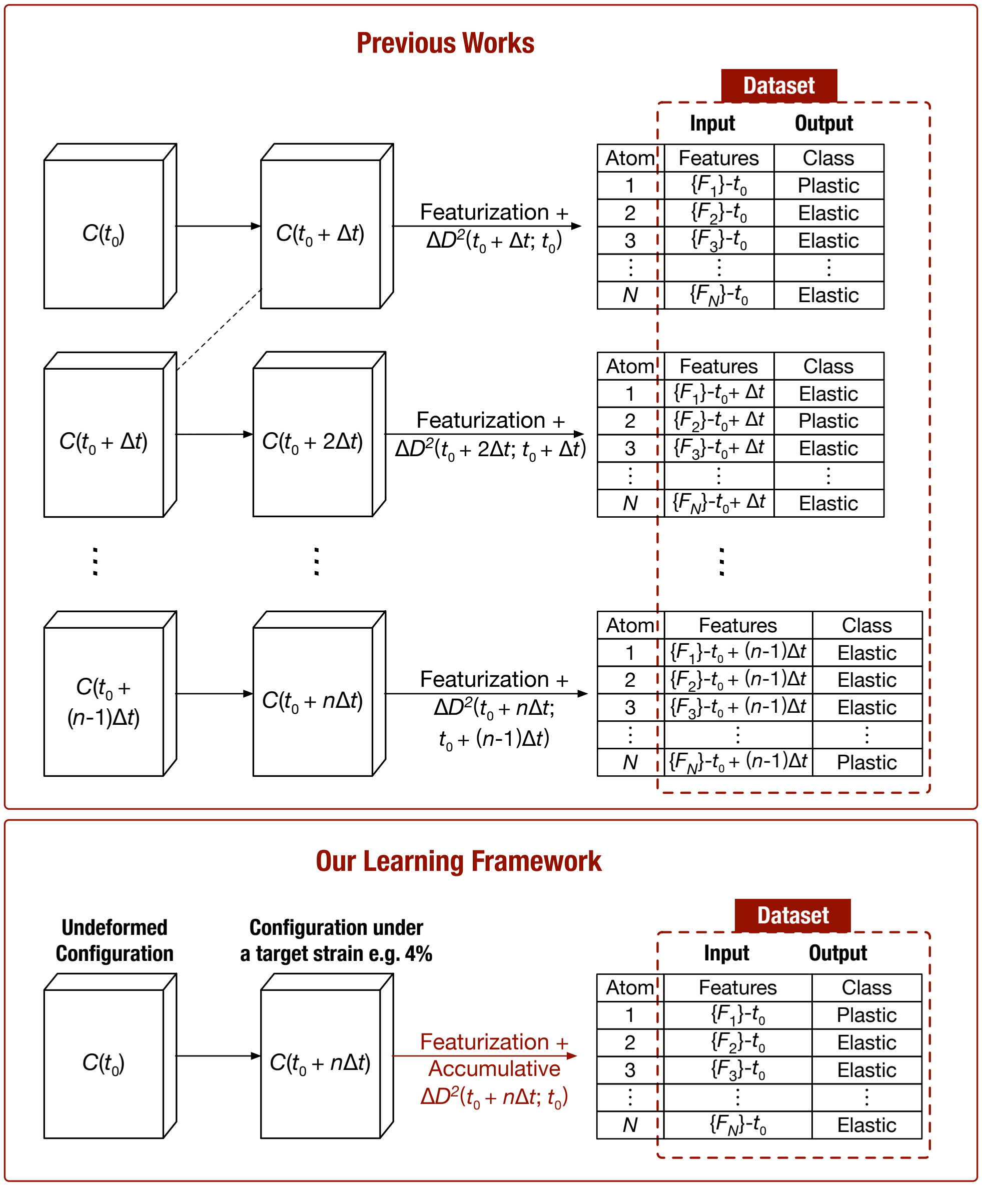

Supplementary Figure 1 | Short- versus long-time plasticity. Illustration of the difference of our machine learning framework with the previous pioneering works by Cubuk and Schoenholz et al.[1–3] Our framework takes the undeformed configuration itself as input and predicts the plastic atoms accumulated within a relatively large strain.

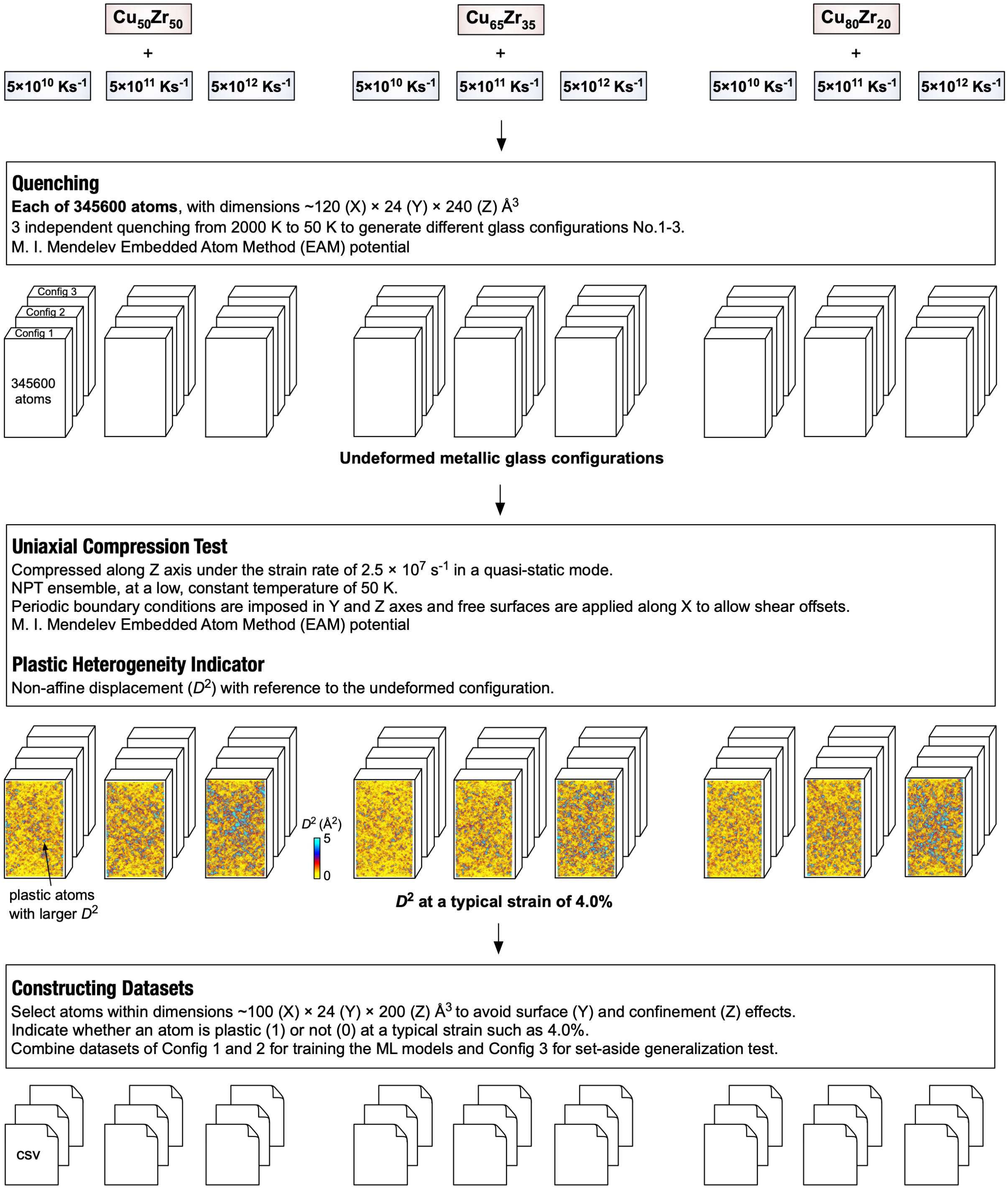

Supplementary Figure 2 | Data generation pathway. The pathway of quenching, uniaxial compression test, plastic heterogeneity indicator calculation and datasets construction in generating atomic-level plastic heterogeneity data of Cu-Zr metallic glasses for machine learning. For $Ni_{62}Nb_{38}$, $Al_{90}Sm_{10}$ and $Fe_{80}P_{20}$, we simulate both tensile and compressive deformation with different strain rates and periodic boundary conditions (Methods).

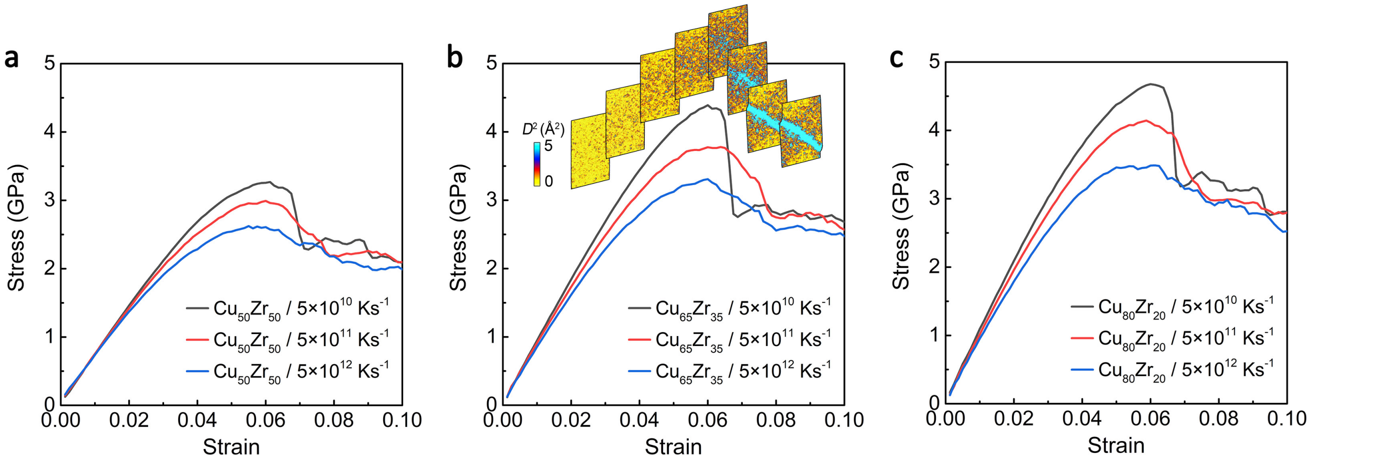

Supplementary Figure 3 | Stress-strain responses. Stress-strain curves of $Cu_{50}Zr_{50}$ (a), $Cu_{65}Zr_{35}$ (b) and $Cu_{80}Zr_{20}$ (c) metallic glasses obtained with quenching rates of 5×10^{10} K s^{-1}, 5×10^{11} K s^{-1} and 5×10^{12} K s^{-1}, respectively. The plastic atoms of at the strain from 2% to 8% with an interval of 1% as well as that at the strain of 6.5% are shown as cyan-blue atoms in the inset picture of (b). The Cu-Zr glass samples (each containing 345600 atoms) are compressed along Z axis under a strain rate of 2.5×10^{7} s^{-1} in a quasi-static mode. Compression is carried out at a low temperature of 50 K. Periodic boundary conditions are imposed in Y and Z axes and free surfaces are applied along X axis to allow offsets.

In contrast to the dislocation-mediated mechanism in crystals, the deformation in metallic glasses (MGs) is largely heterogeneous and difficult to discern. Effective mechanical indicators are needed to detect plastic events in MGs. In this work, we employ non-affine displacement (D^2) as the plastic indicator[4]. To calculate D^2, the locally affine transformation matrix, J_i, that best maps $\{d_{ji}^0\} \rightarrow \{d_{ji}\}$, $\forall j \in N_i^0$, is obtained by minimizing $\sum_{j \in N_i^0} \left| d_{ji}^0 J_i - d_{ji} \right|^2$,

$$J_i = \left(\sum_{j \in N_i^0} d_{ji}^{0T} d_{ji}^0 \right)^{-1} \left(\sum_{j \in N_i^0} d_{ji}^{0T} d_{ji} \right) \quad (1)$$

where d_{ji} and d_{ji}^0 are the vector separation (row vectors) between atom j and i and superscript 0 indicates the reference configuration. Here, j is one of atom i's nearest neighbors, and N_i^0 is the total number of nearest-neighbors of atom i at the reference configuration.

Then, the D^2 for atom i can be defined as the residual of the least square fit:

$$D_i^2 = \frac{1}{N_i^0} \sum_{j \in N_i^0} \left| d_{ji}^0 J_i - d_{ji} \right|^2 \quad (2)$$

Here D^2 is calculated based on a slightly modified code from OVITO[5]. The cutoff in calculating D^2 is set to be 4.5 Å, which falls roughly between the 1st and 2nd peaks in the pair correlation functions of the Cu-Zr glasses. By setting a D^2 threshold of 5.0 Å^2, we can indicate whether an atom is plastic or not at a typical strain such as 4.0%.

In addition to non-affine displacement (D^2), local von-mises strain invariant (η^{mises}) is also a

commonly used plastic indicator[4]. To calculate η^{mises} of for atom i, we also calculate the locally affine transformation matrix, J_i, that best maps $\{d_{ji}^0\} \rightarrow \{d_{ji}\}$, $\forall j \in N_i^0$ by minimizing $\sum_{j \in N_i^0} \left| d_{ji}^0 J_i - d_{ji} \right|^2$, as shown in Equation 2 of the main text. The local Lagrangian strain matrix can then be derived as

$$\eta_i = \frac{1}{2}(J_i J_i^{\mathrm{T}} - I) \tag{3}$$

The local shear invariant η^{mises} of atom i can then be computed as

$$\eta^{\text{mises}} = \sqrt{\eta_{yz}^2 + \eta_{xz}^2 + \eta_{xy}^2 + \frac{(\eta_{yy}^2 - \eta_{zz}^2)^2 + (\eta_{xx}^2 - \eta_{zz}^2)^2 + (\eta_{xx}^2 - \eta_{yy}^2)^2}{6}} \tag{4}$$

The compatibility of D^2 and η^{mises} can be seen in the following Supplementary Figure 4. One can see that the regions with prominent D^2 and η^{mises} are overlapped to a large degree, suggesting that these two plastic indicators capture similar plastic rearrangement regions in the MGs.

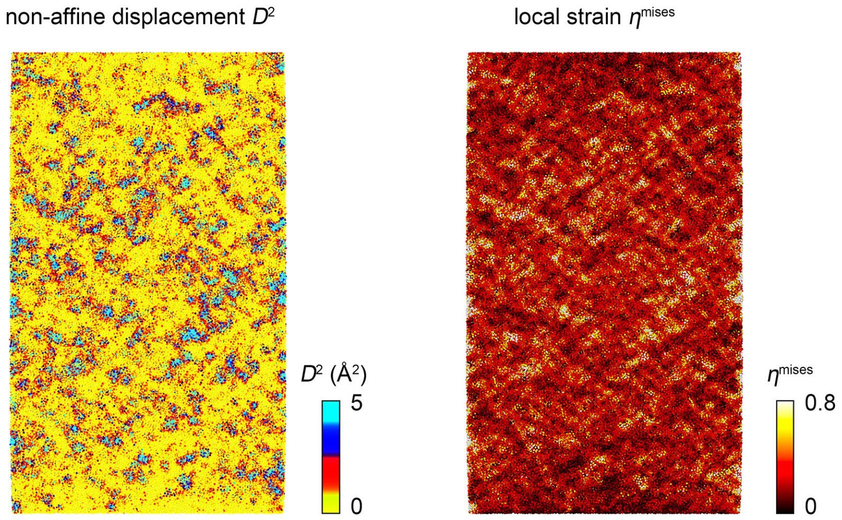

Supplementary Figure 4 | Compatibility of D^2 with other plastic indicators. Compatibility of non-affine displacement (D^2) with another plastic indicator of local shear strain η^{mises}. Distribution of non-affine displacement D^2 (left) and local shear strain η^{mises} at a typical strain of 0.04 of the $Cu_{65}Zr_{35}$ quenched under 5×10^{10} K s^{-1}.

**Supplementary Table 1** | A list of hyperparameters of the gradient boosting decision trees (GBDT) machine learning model that are optimized in this work.	
Hyperparameter	Range
Maximum depth of trees	1, 2, 3, 4, 5
Number of trees	50, 60, …, 300, 350, 400
Minimum samples to split a node	2, 10, 20, …, 100
Minimum samples at a leaf node	1, 10, 20, …, 100
Maximum number of features at each split	n_features, sqrt(n_features), $\log_2$(n_features)

Supplementary Note 2. Recursive feature elimination in Cu-Zr MGs

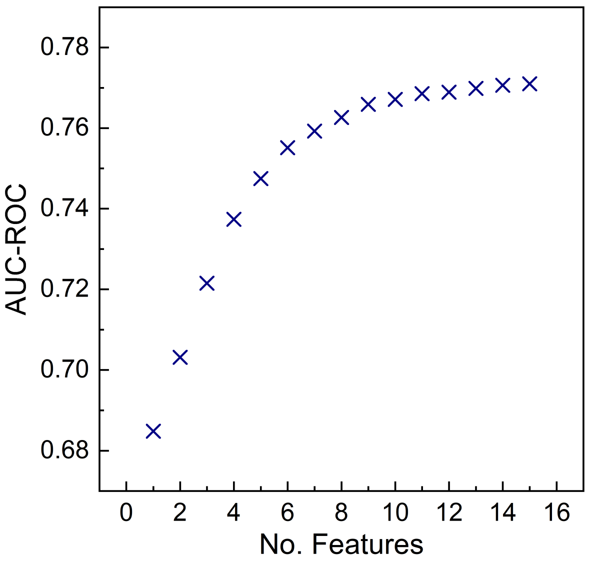

Supplementary Figure 5 | Retained AUC-ROC with varying number of features after recursive feature elimination for $Cu_{65}Zr_{35}$ @ 5 × 10^{10} K s^{-1}.

Supplementary Table 2 | The 15 features for constructing the ML models. The order is in descending order of the 5-fold cross-validation averaged feature importances.

Feature
$\mathrm{MRO_{mean}}\ \mathrm{std}(a_{\mathrm{interstice}})$
$\min(d_{\mathrm{interstice}})$
$\mathrm{MRO_{min}}\ \min(V_{\mathrm{interstice}})$
$\mathrm{mean}(d_{\mathrm{interstice}})$
$\mathrm{MRO_{mean}}\ \min(d_{\mathrm{interstice}})$
$\max(d_{\mathrm{interstice}})$
$\mathrm{mean}(a_{\mathrm{interstice}})$
$\mathrm{MRO_{std}}\ \max(d_{\mathrm{interstice}})$
$\mathrm{MRO_{std}}\ \mathrm{mean}(d_{\mathrm{interstice}})$
$\mathrm{MRO_{mean}}\ \mathrm{std}(d_{\mathrm{interstice}})$
$\mathrm{mean}(d_{\mathrm{interstice\text{-}cutoff}})$
$\mathrm{MRO_{mean}}\ \mathrm{mean}(d_{\mathrm{interstice\text{-}cutoff}})$
$\mathrm{mean}(V_{\mathrm{interstice}})$
$\mathrm{std}(V_{\mathrm{interstice}})$
$\mathrm{std}(d_{\mathrm{interstice\text{-}cutoff}})$

As stated in the Methods, in this work, we use AUC-ROC as the scoring metric, as it best suits our target of this study. In terms of the 3rd point (AUC-ROC is robust with the imbalanced data), Supplementary Figure 6 illustrates the AUC-ROCs of data with different degrees of imbalance: i) an imbalanced dataset of an unseen glass configuration without any sampling; ii) a balanced test set after equal undersampling and train-test (80:20) split; iii) an imbalanced dataset combining the balanced test set of ii) with all the unsampled majority samples.

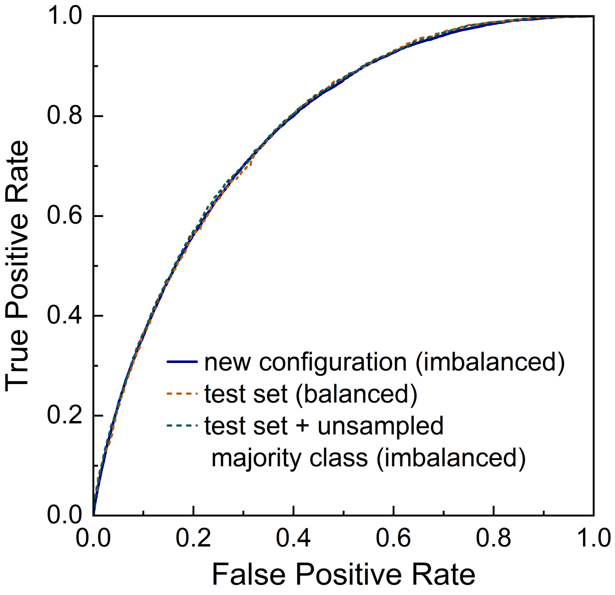

Supplementary Figure 6 | AUC insensitive to the imbalanced degree of datasets.

Supplementary Note 3. Symmetry functions and other existing features

In previous works, symmetry functions, originally proposed to represent the potential energy landscape of systems[6,7], have been successfully employed to featurize the local environments in various disordered solids and liquids.[1–3] The symmetry functions are individually less descriptive but can collectively provide a complete and unbiased description of the structure. As presented in the Methods, we follow the settings of r, ξ, λ, and ζ in previous works[1–3] to calculate the symmetry functions for each atom in our studied MGs. Specifically, for an atom i in Cu-Zr MGs, we derive 100 radial functions (50 for i-Cu and 50 for i-Zr) by varying r from 0 to 5.0 × Cu-Cu equilibrium distance (sum of metallic radii) in increments of 0.1 × Cu-Cu equilibrium distance, and 66 angular functions (22 for Cu-i-Cu, Cu-i-Zr and Zr-i-Zr, respectively) by using 22 sets of ξ, λ, and ζ for each atom. Please refer to the original papers[1–3] for details of parameter settings.

We then take the 166 features as input and train a GBDT model on the same datasets and cross-validation (CV) splits with our interstice features. The hyperparameters of the GBDT model are also optimized based on Supplementary Table 1. After optimizing a model on the data sampled from two glass configurations, the model is generalized to the unseen configuration set-aside for tests (Methods). Supplementary Table 3 shows the results of the ML models on the unseen glass configuration when using the symmetry functions as input. The results of using all the 80 interstice features or the 15 interstice features after feature reduction are also displayed for comparison.

Supplementary Table 3 | AUC-ROCs when using our interstice distribution features or the symmetry functions to fit a GBDT model for Cu-Zr MGs. The AUC-ROCs are obtained by generalizing the models fitted on the same sampled data and CV splits to the unseen glass configuration.

Comp./ Quenching Rate	Our work (80 features)	Our work (15 features)	Symmetry function (166 features)
$Cu_{65}Zr_{35}$ / 5×10^{10} K s^{-1}	0.772	0.771	0.751
$Cu_{50}Zr_{50}$ / 5×10^{10} K s^{-1}	0.733	0.733	0.716
$Cu_{80}Zr_{35}$ / 5×10^{10} K s^{-1}	0.766	0.766	0.757

Supplementary Table 4 | Representative short-range order (SRO) feature sets.

<table>
<tr><th>Features</th><th>Description</th><th>Count</th></tr>
<tr><td>$CN_{Voro/Dist}$</td><td>Number of neighboring atoms determined by Voronoi tessellation analysis[8] or within a cutoff distance.</td><td>2</td></tr>
<tr><td>Voronoi $idx_{3...7}$</td><td>$\{n_i\}$ where n_i denotes the number of i-edged facets (i in the range of 3-7) of the Voronoi polyhedra[8].</td><td>5</td></tr>
<tr><td>Characteristic motifs</td><td>One-hot encoded signatures of whether a cluster belongs to characteristic motifs e.g. <0,0,12,0,0>, <0,0,12,4,0>, <0,0,12,0,0>||<0,0,12,4,0> or Frank-Kasper-type clusters[9].</td><td>4</td></tr>
<tr><td>Volume metrics</td><td>Cluster packing efficiency[12], atomic packing efficiency[13], and the ratio of atomic volume to the Voronoi polyhedron volume around each site.</td><td>3</td></tr>
<tr><td>i-fold symm $idx_{3...7}$</td><td>$n_i / \sum_{i=3}^{7} n_i$ where n_i is Voronoi index (i in the range of 3-7), reflecting the strength of i-fold symmetry in local sites[14].</td><td>5</td></tr>
<tr><td>Weighted i-fold symm $idx_{3...7}$</td><td>Weighted i-fold symmetry indices using Voronoi facet areas.</td><td>5</td></tr>
<tr><td>BOOP $q_{4...10\text{-}Voro/Dist}$ and $w_{4...10\text{-}Voro/Dist}$</td><td>Lowest-order and higher-order rotation-invariant q_l (l = 4, 6, 8 and 10) of the lth moment in a multipole expansion of the bond vector distribution on a unit sphere[11].</td><td>16</td></tr>
<tr><td>$CSRO_{Voro/Dist}$</td><td>Element type, the number and the deviation of local chemistry with nominal composition (Warren-Cowley parameters[15,16]).</td><td>9</td></tr>
<tr><td>Subtotal</td><td>Candidate SRO features for an atomic site.</td><td>49</td></tr>
</table>

In addition to symmetry functions, there are also some conventional short-range order (SRO) structural signatures in the field of MGs. Supplementary Table 4 lists some typical structural signatures proposed in the past studies[8–16]. These signatures can readily be derived from the structure itself, without requiring knowledge of detailed interatomic interactions, and are thus pure structural signatures as well. In Supplementary Table 4, the first 7 feature sets are geometrical features (GSRO) and the last feature set is the chemical (CSRO) feature set.

We then featurize the atoms in Cu-Zr MGs with these existing SRO features, and try augmenting our features with these SRO features. However, the AUCs only have a minimal change, as summarized in Supplementary Table 5.

Supplementary Table 5 | AUC-ROCs of the Cu-Zr MGs by augmenting our interstice distribution features with existing SRO features to fit a GBDT model. GSRO features represent geometrical SRO features and CSRO features indicate chemical SRO features.

Comp./ Quenching Rate	Our + GSRO (120 features)	Our + CSRO (89 features)	Our + SRO (129 features)
$Cu_{65}Zr_{35}$ / 5×10^{10} K s^{-1}	0.771	0.771	0.771
$Cu_{50}Zr_{50}$ / 5×10^{10} K s^{-1}	0.734	0.733	0.734
$Cu_{80}Zr_{35}$ / 5×10^{10} K s^{-1}	0.767	0.767	0.767

We also tried generating the corresponding medium-range order (MRO) versions of the existing SRO features, following the coarse-graining technique described in the main text (e.g. Equation 1). Supplementary Table 6 lists the MRO feature sets we have generated in this work. Similarly, they can be considered as medium-range geometrical features (GMRO) or chemical features (GMRO).

Supplementary Table 7 summarizes the results by augmenting our interstice distribution features with these MRO features. Similarly, they contribute little to the decision making of the obtained ML models.

Supplementary Table 6 | Medium-range order (MRO) feature sets generated by an automatic neighboring coarse-grained method.

Features	Statistical Types	Count
MRO $CN_{Voro/Dist}$	mean, std, min and max of $CN_{Voro/Dist}$.	8
MRO Voronoi $idx_{3...7}$	mean, std, min and max of Voronoi $indices_{3...7}$.	20
MRO Characteristic motifs	sum, mean, std of Characteristic motifs.	12
MRO Volume metrics	mean, std, min and max of Volume metrics.	12
MRO Avg. i-fold symm $idx_{3...7}$	$\sum_{m=0}^{NN} n_i^m / \sum_{m=0}^{NN} \sum_{i=3}^{7} n_i^m$, where n_i denotes the number of i-edged facets of the Voronoi polyhedra and m iterates over each neighbor.	5
MRO i-fold symm $idx_{3...7}$	mean, std, min and max of i-fold symm $idx_{3...7}$.	20
MRO Weighted i-fold symm $idx_{3...7}$	mean, std, min and max of Weighted i-fold symm $idx_{3...7}$.	20
MRO BOOP $q_{4...10\text{-Voro/Dist}}$ and $w_{4...10\text{-Voro/Dist}}$	mean, std, min and max of BOOP $q_{4...10\text{-Voro/Dist}}$ and $w_{4...10\text{-Voro/Dist}}$.	64
Coarse-grained BOOP $q_{4...10\text{-Voro/Dist}}$ and $w_{4...10\text{-Voro/Dist}}$	Coarse-grained[17] lowest-order and higher-order rotation-invariant $\overline{q}_l$ (l = 4, 6, 8 and 10).	16
$CMRO_{Voro/Dist}$	mean, std, min and max of $CSRO_{Voro}$ except for the element type.	32
Subtotal	Candidate MRO features for an atomic site.	209

Supplementary Table 7 | AUC-ROCs of the Cu-Zr MGs by augmenting our interstice distribution features with the generated MRO features to fit a GBDT model. GMRO features represent geometrical MRO features and CMRO features indicate chemical MRO features.

Comp./ Quenching Rate	Our + GMRO (257 features)	Our + CMRO (112 features)	Our + MRO (289 features)
$Cu_{65}Zr_{35}$ / 5×10^{10} K s^{-1}	0.771	0.772	0.772
$Cu_{50}Zr_{50}$ / 5×10^{10} K s^{-1}	0.733	0.734	0.733
$Cu_{80}Zr_{35}$ / 5×10^{10} K s^{-1}	0.766	0.767	0.767

Supplementary Note 4. Calibration curves of GBDT and Linear SVC models

The "quench-in softness" (QS) proposed in this work is basically the class probability estimate from the GBDT models. The idea is that when performing classification, we want to not only obtain the class label but also have a probability of the respective class that gives us some sense of confidence on the predictions[18].

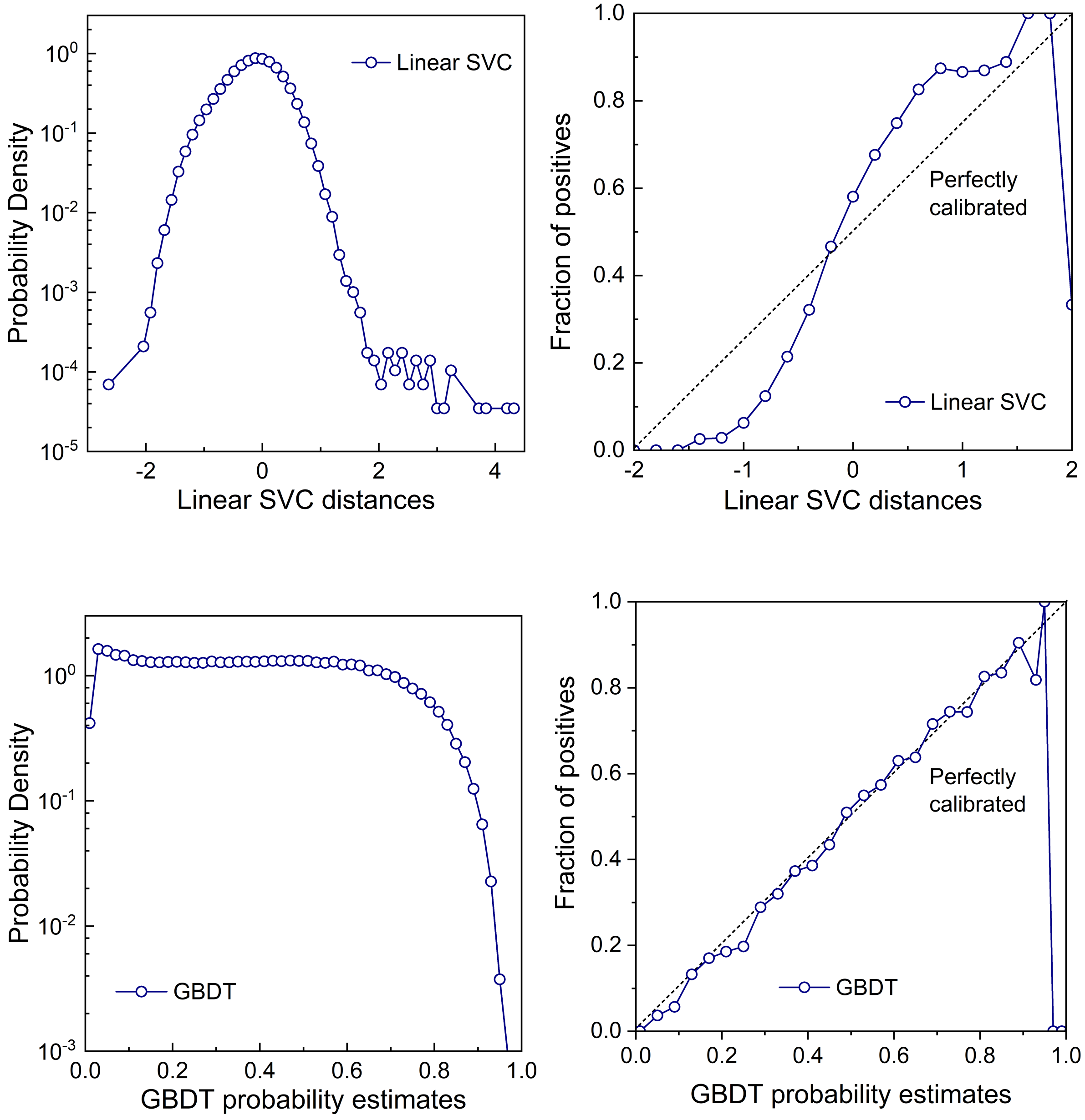

Supplementary Figure 7 | Probability density distributions and calibration curves of Linear SVC models (upper panel) and our GBDT models (lower panel) using the same interstice distribution features and on the same data of the $Cu_{65}Zr_{35}$ @ 5×10^{10} K s^{-1}. The calibration curves are plotted on the resampled balanced test set.

In previous works, researchers have defined the distances to the separating Linear SVC hyperplane as "softness"[2,3]. The distances can also serve as a continuous metric to reflect the propensity to be one class, yet the distances are unbounded and often require calibration to transform the unbounded distances into probability estimates (bounded in [0, 1]). Applying min-max scaling of the SVC distances to [0, 1] often yields bad calibration, and the calibration curve often shows sigmoid curve, which is typical for maximum-margin methods that focus on hard-to-classify samples that are close to the decision boundary (support vectors)[18]. GBDT are also prone to have bad estimates, however, here we verify that our GBDT probability estimates are well-calibrated, largely attributed to the shallow trees and small number of trees used in our work.

As an example, Supplementary Figure 7 shows the comparison of probability density distributions and calibration curves of Linear SVC models and our GBDT models if fitted on same interstice features and same data of $Cu_{65}Zr_{35}$. The calibration curves are plotted on the undersampled balanced test set. We see that the calibration curve of raw SVM distances is quite sigmoid-like, deviating much from the perfect calibrated curve (diagonal line), while the calibration curve of our GBDT probability estimates are well-calibrated. Well-calibrated probability estimates can then be directly interpreted as the confidence level of classification. Readers can refer to the website in Ref. [18] for more examples of calibration. If one is interested in transforming the SVM distances into well-calibrated probability estimates, the parametric approach based on Platt's sigmoid model[19] and the non-parametric approach based on isotonic regression[20] could be helpful for this end.

Supplementary Note 5. ML results of Ni-Nb/Al-Sm/Fe-P MGs

Supplementary Table 8 | Classifying plastic atoms accumulated up to the strain of 4.0% of $Ni_{62}Nb_{38}$, $Al_{90}Sm_{10}$, and $Fe_{80}P_{20}$ based on the undeformed structure features.

Comp./ Quenching Rate	Deformation Mode/ Strain Rate	Dataset Info./ D^2 threshold and sampled atoms	Our work (80 features)	Symmetry functions (166 features)
$Al_{90}Sm_{10}$/ 5×10^{10} K s^{-1}	Compressive + ppp 2.5×10^7 s^{-1}	79295 Al atoms D^2>7.0 6310 atoms	0.771	0.758
	Tensile + ppp 2.5×10^7 s^{-1}	79295 Al atoms D^2>5.0 6746 atoms	0.761	0.752
	Tensile + ppp 1×10^8 s^{-1}	79295 Al atoms D^2>5.0 6292 atoms	0.770	0.760
	Compressive + pps 2.5×10^7 s^{-1}	79295 Al atoms D^2>8.0 6832 atoms	0.772	0.761
	Tensile + pps 2.5×10^7 s^{-1}	79295 Al atoms D^2>5.0 6244 atoms	0.763	0.755
	Tensile + pps 1×10^8 s^{-1}	79295 Al atoms D^2>5.0 5794 atoms	0.775	0.768
$Ni_{62}Nb_{38}$/ 5×10^{10} K s^{-1}	Compressive + ppp 2.5×10^7 s^{-1}	55635 Ni atoms D^2>3.0 3692 atoms	0.764	0.733
	Tensile + ppp 2.5×10^7 s^{-1}	55635 Ni atoms D^2>3.0 3236 atoms	0.773	0.743
	Tensile + ppp 1×10^8 s^{-1}	55635 Ni atoms D^2>3.0 3150 atoms	0.763	0.736
$Fe_{80}P_{20}$/ 5×10^{10} K s^{-1}	Compressive + ppp 2.5×10^7 s^{-1}	69223 Fe atoms D^2>2.5 3776 atoms	0.737	0.756
	Compressive + ppp 2.5×10^7 s^{-1}	69223 Fe atoms D^2>3.0 2654 atoms	0.741	0.768

Supplementary Note 6. Generalization results of ML models

Supplementary Table 9 | Typical generalization results of ML models using our interstice distribution features or symmetry functions on the following scenarios: i) same composition and different quenching rate; ii) different composition and same quenching rate; iii) different composition and different quenching rate; and iv) different chemical system. In the table, G and F are short for generalized and fitted scores, respectively.

From: glass A (1st row)<br>To: glass B (2nd row)	Our work		Symmetry Functions	
	Fitted	Generalized (G - F)	Fitted	Generalized (G - F)
**Same composition, Different quenching rate**				
$Cu_{65}Zr_{35}/5\times10^{11}$ Ks^{-1}<br>$Cu_{65}Zr_{35}/5\times10^{10}$ Ks^{-1}	0.771	0.771<br>(0)	0.751	0.751<br>(0)
$Cu_{65}Zr_{35}/5\times10^{12}$ Ks^{-1}<br>$Cu_{65}Zr_{35}/5\times10^{10}$ Ks^{-1}	0.771	0.771<br>(0)	0.751	0.750<br>(-0.001)
$Cu_{50}Zr_{50}/5\times10^{11}$ Ks^{-1}<br>$Cu_{50}Zr_{50}/5\times10^{10}$ Ks^{-1}	0.733	0.733<br>(0)	0.717	0.717<br>(0)
$Cu_{50}Zr_{50}/5\times10^{12}$ Ks^{-1}<br>$Cu_{50}Zr_{50}/5\times10^{10}$ Ks^{-1}	0.733	0.733<br>(-0.001)	0.717	0.717<br>(0)
$Cu_{80}Zr_{20}/5\times10^{11}$ Ks^{-1}<br>$Cu_{80}Zr_{20}/5\times10^{10}$ Ks^{-1}	0.766	0.766<br>(0)	0.756	0.756<br>(0)
$Cu_{80}Zr_{20}/5\times10^{12}$ Ks^{-1}<br>$Cu_{80}Zr_{20}/5\times10^{10}$ Ks^{-1}	0.766	0.766<br>(0)	0.756	0.756<br>(0)
**Different composition, Same quenching rate**				
$Cu_{50}Zr_{50}/5\times10^{10}$ Ks^{-1}<br>$Cu_{65}Zr_{35}/5\times10^{10}$ Ks^{-1}	0.771	0.771<br>(0)	0.751	0.739<br>(-0.012)
$Cu_{80}Zr_{20}/5\times10^{10}$ Ks^{-1}<br>$Cu_{65}Zr_{35}/5\times10^{10}$ Ks^{-1}	0.771	0.769<br>(-0.002)	0.751	0.729<br>(-0.022)
$Cu_{65}Zr_{35}/5\times10^{10}$ Ks^{-1}<br>$Cu_{50}Zr_{50}/5\times10^{10}$ Ks^{-1}	0.733	0.728<br>(-0.005)	0.716	0.704<br>(-0.012)
$Cu_{80}Zr_{20}/5\times10^{10}$ Ks^{-1}<br>$Cu_{50}Zr_{50}/5\times10^{10}$ Ks^{-1}	0.733	0.721<br>(-0.012)	0.716	0.655<br>(-0.061)
$Cu_{65}Zr_{35}/5\times10^{10}$ Ks^{-1}<br>$Cu_{80}Zr_{20}/5\times10^{10}$ Ks^{-1}	0.766	0.759<br>(-0.007)	0.757	0.709<br>(-0.048)

$Cu_{50}Zr_{50}$/5×10^{10} Ks^{-1} $Cu_{80}Zr_{20}$/5×10^{10} Ks^{-1}	0.766	0.759 (-0.006)	0.757	0.648 (-0.109)
**Different composition, Different quenching rate**				
$Cu_{50}Zr_{50}$/5×10^{12} Ks^{-1} $Cu_{65}Zr_{35}$/5×10^{10} Ks^{-1}	0.771	0.771 (0)	0.751	0.745 (-0.006)
$Cu_{80}Zr_{20}$/5×10^{12} Ks^{-1} $Cu_{65}Zr_{35}$/5×10^{10} Ks^{-1}	0.771	0.770 (-0.001)	0.751	0.749 (-0.002)
$Cu_{65}Zr_{35}$/5×10^{12} Ks^{-1} $Cu_{50}Zr_{50}$/5×10^{10} Ks^{-1}	0.733	0.731 (-0.002)	0.716	0.711 (-0.005)
$Cu_{80}Zr_{20}$/5×10^{12} Ks^{-1} $Cu_{50}Zr_{50}$/5×10^{10} Ks^{-1}	0.733	0.722 (-0.011)	0.716	0.691 (-0.025)
$Cu_{65}Zr_{35}$/5×10^{12} Ks^{-1} $Cu_{80}Zr_{20}$/5×10^{10} Ks^{-1}	0.766	0.762 (-0.004)	0.757	0.746 (-0.011)
$Cu_{50}Zr_{50}$/5×10^{12} Ks^{-1} $Cu_{80}Zr_{20}$/5×10^{10} Ks^{-1}	0.766	0.760 (-0.006)	0.757	0.666 (-0.091)
**Different system**				
$Cu_{65}Zr_{35}$/5×10^{10} Ks^{-1} $Ni_{62}Nb_{38}$/5×10^{10} Ks^{-1}	0.764	0.755 (-0.009)	0.733	0.727 (-0.006)
$Cu_{50}Zr_{50}$/5×10^{10} Ks^{-1} $Ni_{62}Nb_{38}$/5×10^{10} Ks^{-1}	0.764	0.754 (-0.010)	0.733	0.706 (-0.027)
$Cu_{80}Zr_{20}$/5×10^{10} Ks^{-1} $Ni_{62}Nb_{38}$/5×10^{10} Ks^{-1}	0.764	0.757 (-0.007)	0.733	0.721 (-0.012)
$Cu_{65}Zr_{35}$/5×10^{10} Ks^{-1} $Al_{90}Sm_{10}$/5×10^{10} Ks^{-1}	0.771	0.765 (-0.006)	0.758	0.688 (-0.070)
$Cu_{50}Zr_{50}$/5×10^{10} Ks^{-1} $Al_{90}Sm_{10}$/5×10^{10} Ks^{-1}	0.771	0.769 (-0.002)	0.758	0.648 (-0.110)
$Cu_{80}Zr_{20}$/5×10^{10} Ks^{-1} $Al_{90}Sm_{10}$/5×10^{10} Ks^{-1}	0.771	0.768 (-0.003)	0.758	0.754 (-0.004)
$Cu_{65}Zr_{35}$/5×10^{10} Ks^{-1} $Fe_{80}P_{20}$/5×10^{10} Ks^{-1}	0.737	0.652 (-0.085)	0.756	0.573 (-0.183)
$Cu_{50}Zr_{50}$/5×10^{10} Ks^{-1} $Fe_{80}P_{20}$/5×10^{10} Ks^{-1}	0.737	0.643 (-0.094)	0.756	0.527 (-0.229)
$Cu_{80}Zr_{20}$/5×10^{10} Ks^{-1} $Fe_{80}P_{20}$/5×10^{10} Ks^{-1}	0.737	0.648 (-0.089)	0.756	0.628 (-0.128)

Supplementary Table 10 | The feature ranking for $Fe_{80}P_{20}$. The order is in descending order of the 5-fold cross-validation averaged feature importances. The ranking of each feature for $Cu_{65}Zr_{35}$ is extracted from Supplementary Table 2 and listed for comparison.

Feature	Feature Ranking for $Cu_{65}Zr_{35}$
$\max(d_{\text{interstice}})$	6
$\text{mean}(d_{\text{interstice-cutoff}})$	11
$\min(d_{\text{interstice}})$	2
$\text{mean}(a_{\text{interstice}})$	7
$\text{MRO}_{\text{min}}\ \min(V_{\text{interstice}})$	3
$\text{MRO}_{\text{mean}}\ \text{mean}(d_{\text{interstice-cutoff}})$	12
$\text{mean}(d_{\text{interstice}})$	4
$\text{MRO}_{\text{std}}\ \text{mean}(d_{\text{interstice}})$	9
$\text{MRO}_{\text{mean}}\ \text{std}(a_{\text{interstice}})$	1
$\text{std}(V_{\text{interstice}})$	14
$\text{MRO}_{\text{mean}}\ \min(d_{\text{interstice}})$	5
$\text{MRO}_{\text{mean}}\ \text{std}(d_{\text{interstice}})$	10
$\text{MRO}_{\text{std}}\ \max(d_{\text{interstice}})$	8
$\text{std}(d_{\text{interstice-cutoff}})$	15
$\text{mean}(V_{\text{interstice}})$	13

Supplementary Note 7. Interpreting the feature sets

As described in the main text, we benchmarked the individual predictive power of 18 feature sets to provide a quantitative picture on their correlation with plastic heterogeneity (Figure 5a). In this section, we further interpret the ML model obtained with each feature set to show the dependence of deformation probability on these site features (Supplementary Figures 8 – 25). For each feature set, two-dimensional (2-D) and one-dimensional (1-D) partial dependence plots (PDP) are shown for the top-4 features with the highest feature importance in each feature set (except for the $CN_{Voro/Dist}$ and Volume metrics feature sets with 2 and 3 features, respectively). Please refer to Methods or Supplementary Tables 4 and 6 for details of the SRO and MRO feature sets.

Short-range order (SRO) feature sets

1) $CN_{Voro/Dist}$

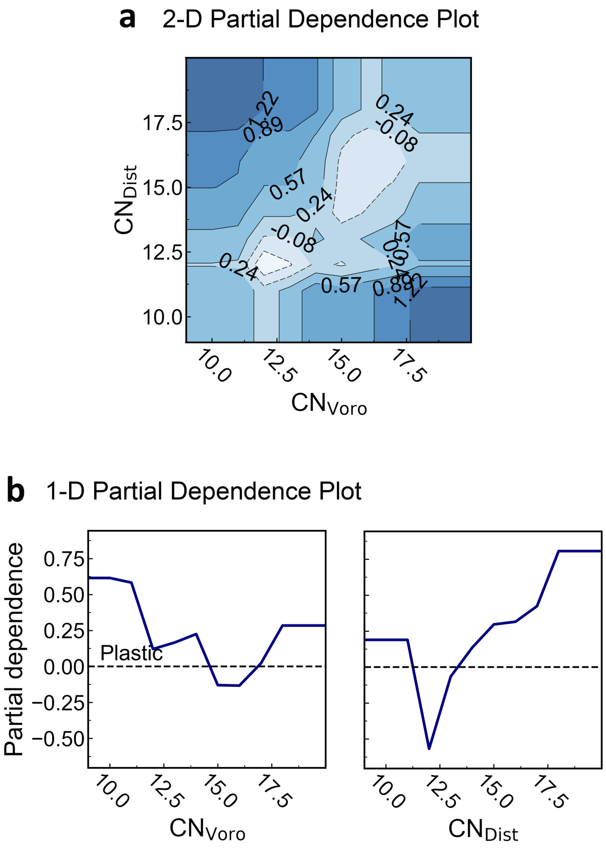

Supplementary Figure 8 | Interpreting the ML model fitted with $CN_{Voro/Dist}$. (a) Two-dimensional (2-D) and (b) one-dimensional (1-D) partial dependence plots (PDPs) of 2 features in the ML model are exhibited.

2) Voronoi $idx_{3...7}$

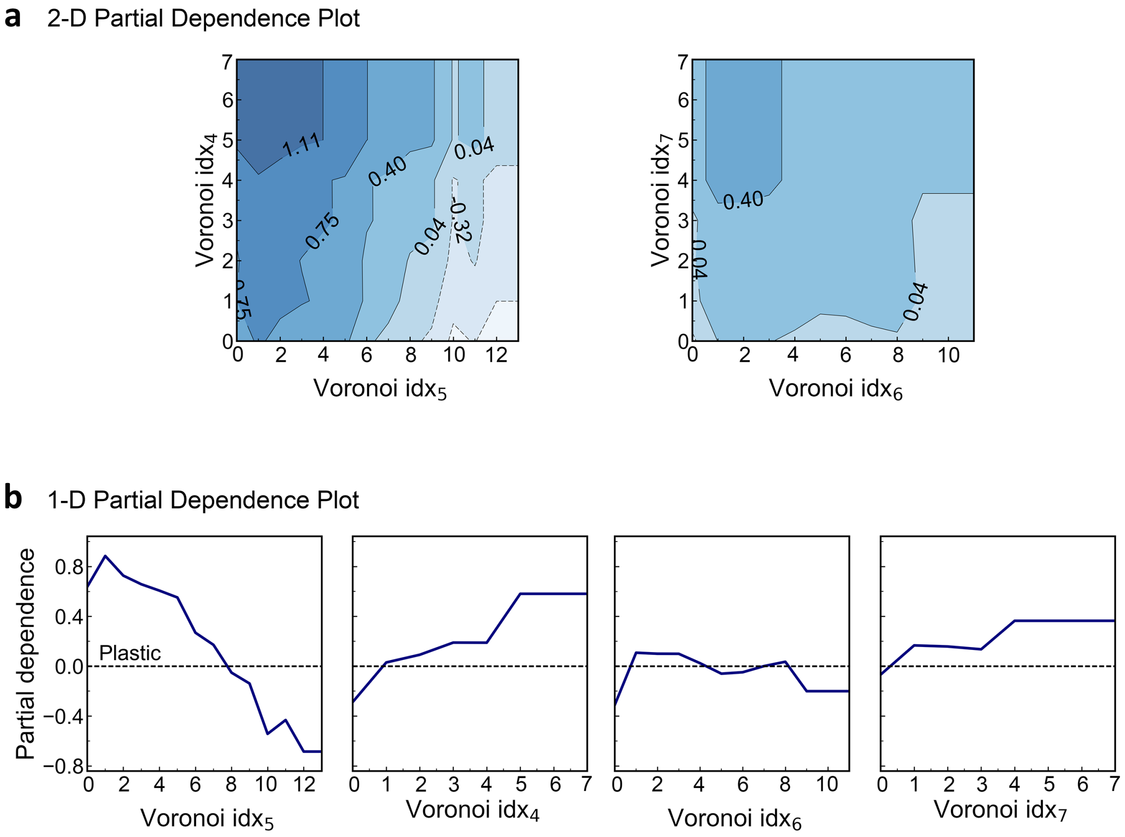

Supplementary Figure 9 | Interpreting the ML model fitted with Voronoi $idx_{3...7}$. (a) Two-dimensional (2-D) and (b) one-dimensional (1-D) partial dependence plots (PDPs) of top 4 features in the ML model are exhibited.

3) Characteristic motifs

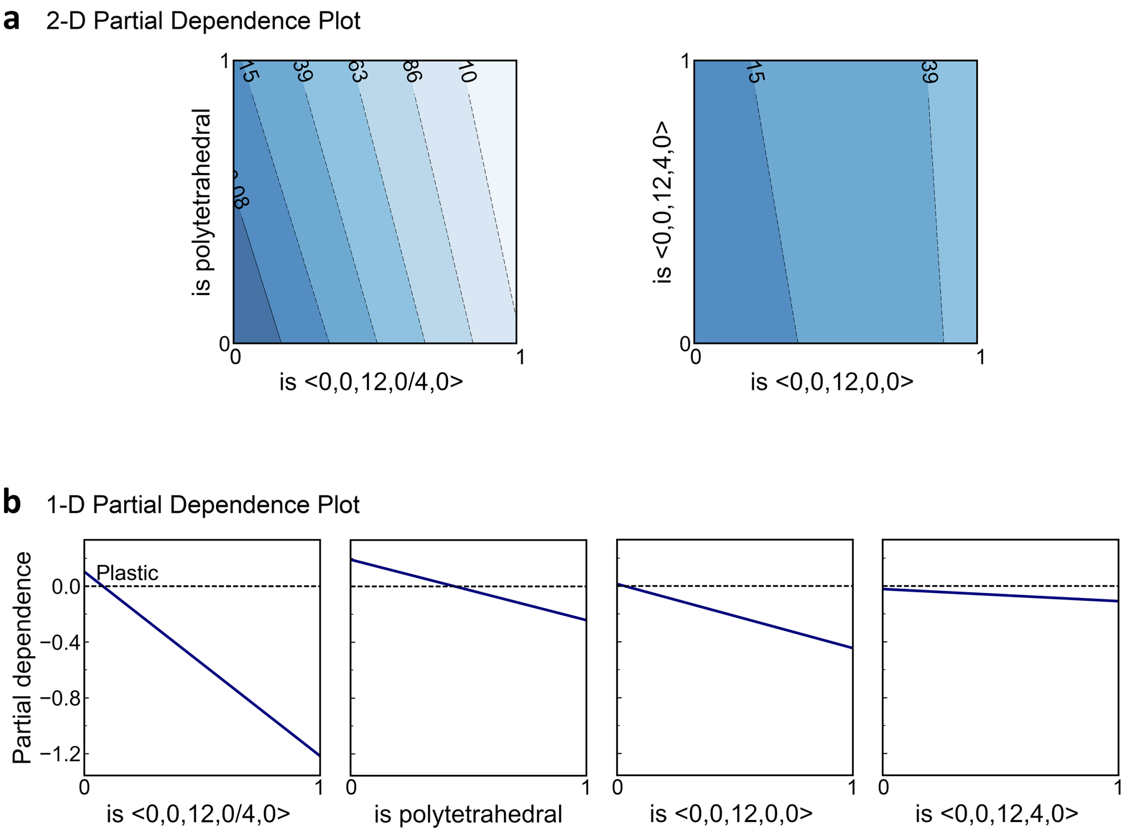

Supplementary Figure 10 | Interpreting the ML model fitted with Characteristic motifs. (a) Two-dimensional (2-D) and (b) one-dimensional (1-D) partial dependence plots (PDPs) of top 4 features in the ML model are exhibited.

4) Volume metrics

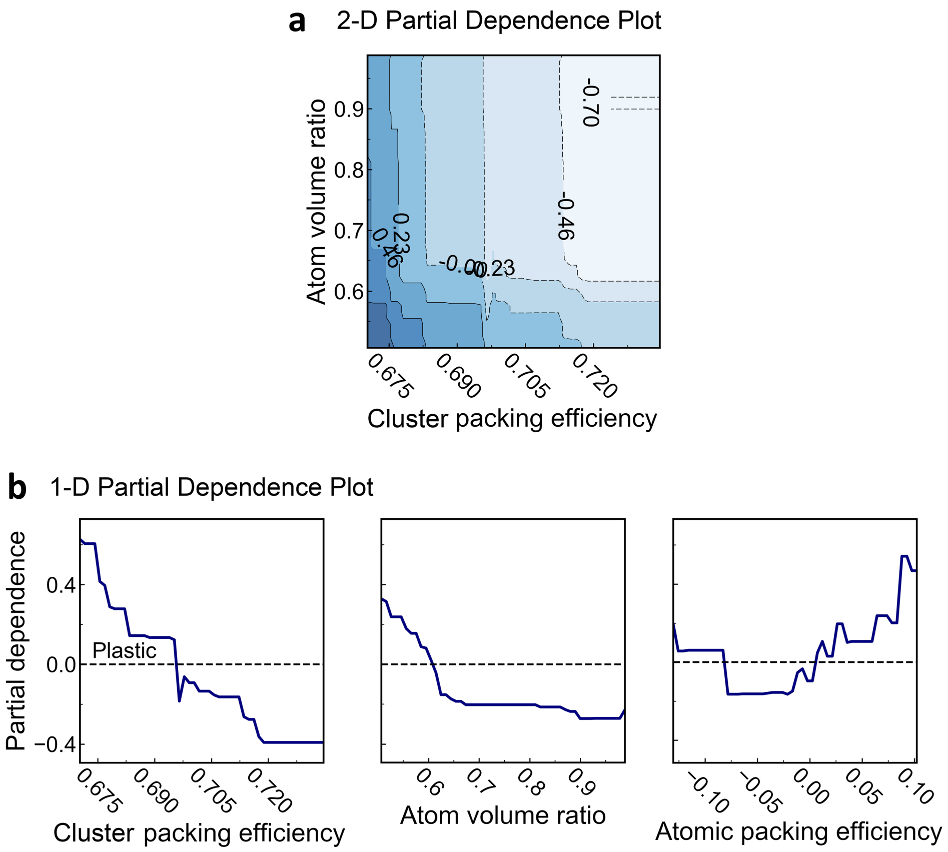

Supplementary Figure 11 | Interpreting the ML model fitted with Volume metrics. (a) Two-dimensional (2-D) of the top 2 features and (b) one-dimensional (1-D) partial dependence plots (PDPs) of the 3 features in the ML model are exhibited.

5) *i*-fold symmetry idx$_{3\dots7}$

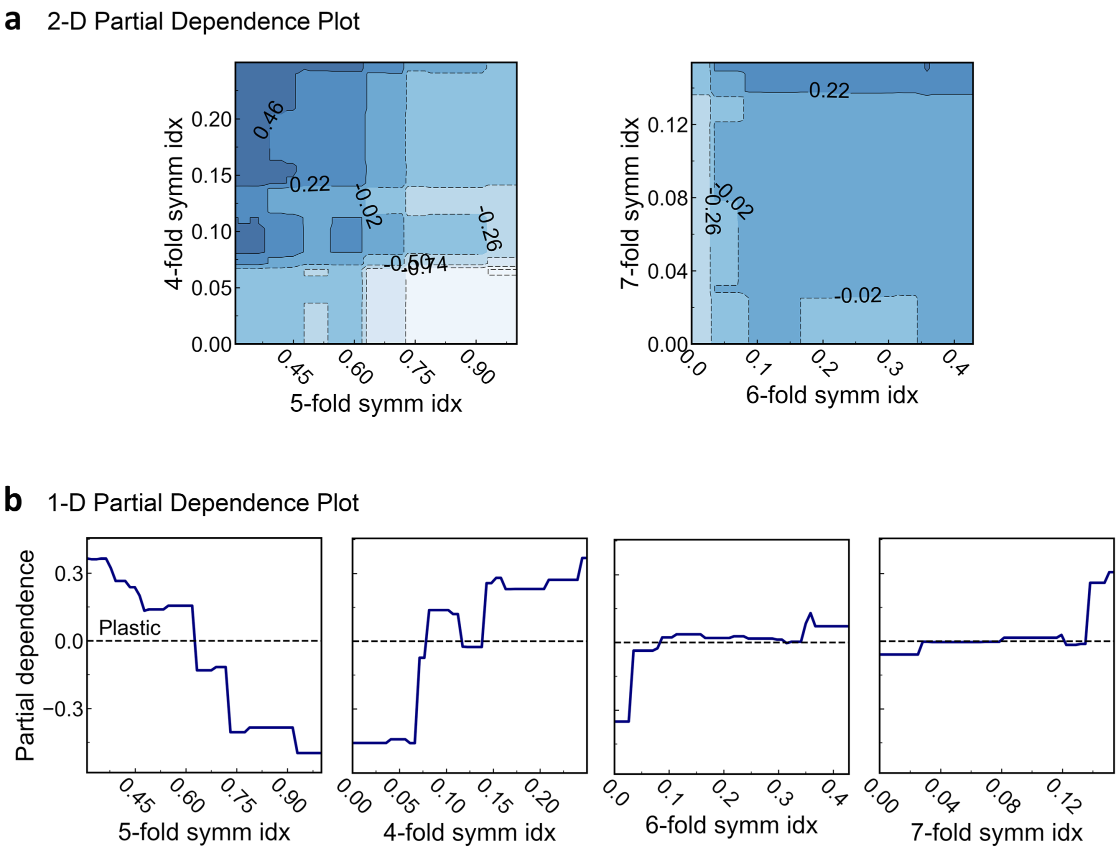

Supplementary Figure 12 | Interpreting the ML model fitted with *i*-fold symmetry idx$_{3\dots7}$. (a) Two-dimensional (2-D) and (b) one-dimensional (1-D) partial dependence plots (PDPs) of top 4 features in the ML model are exhibited.

6) Weighted *i*-fold symmetry idx$_{3...7}$

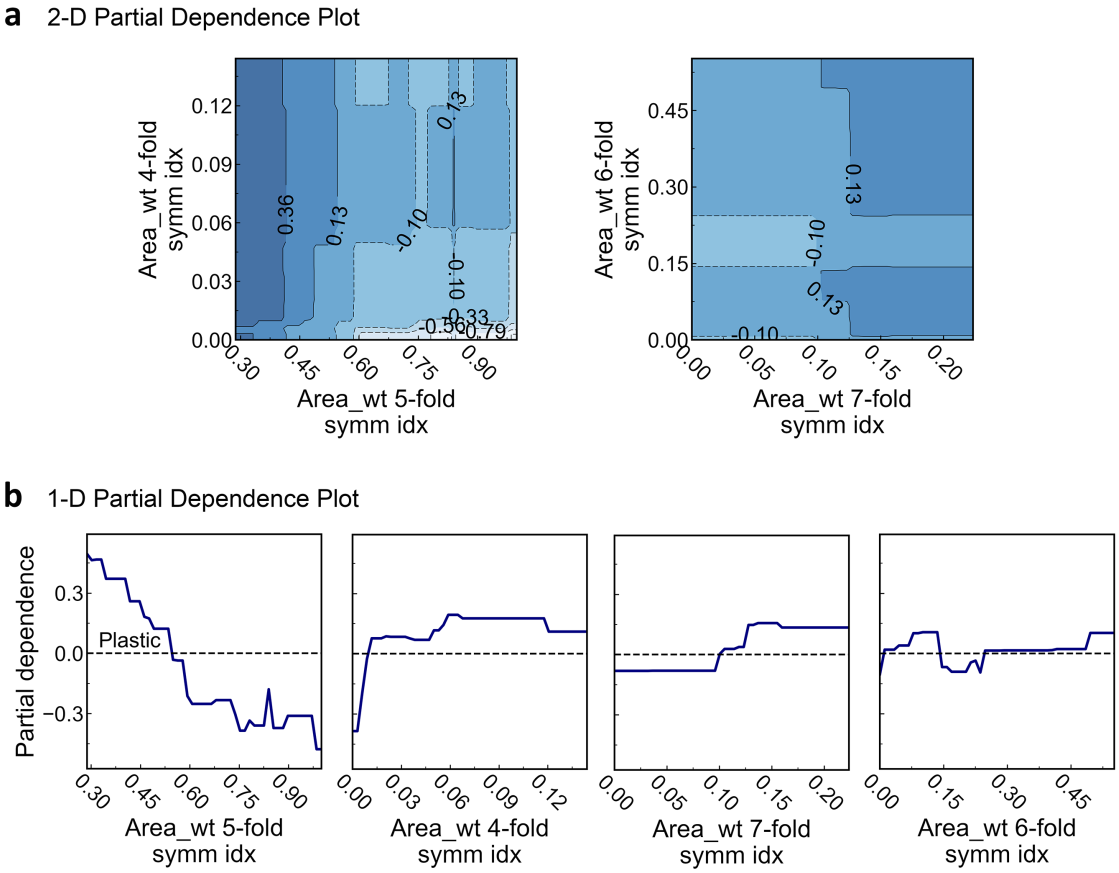

Supplementary Figure 13 | Interpreting the ML model fitted with Weighted *i*-fold symmetry idx$_{3...7}$. (a) Two-dimensional (2-D) and (b) one-dimensional (1-D) partial dependence plots (PDPs) of top 4 features in the ML model are exhibited.

7) BOOP $q_{4\ldots10\text{-Voro/Dist}}$ and $w_{4\ldots10\text{-Voro/Dist}}$

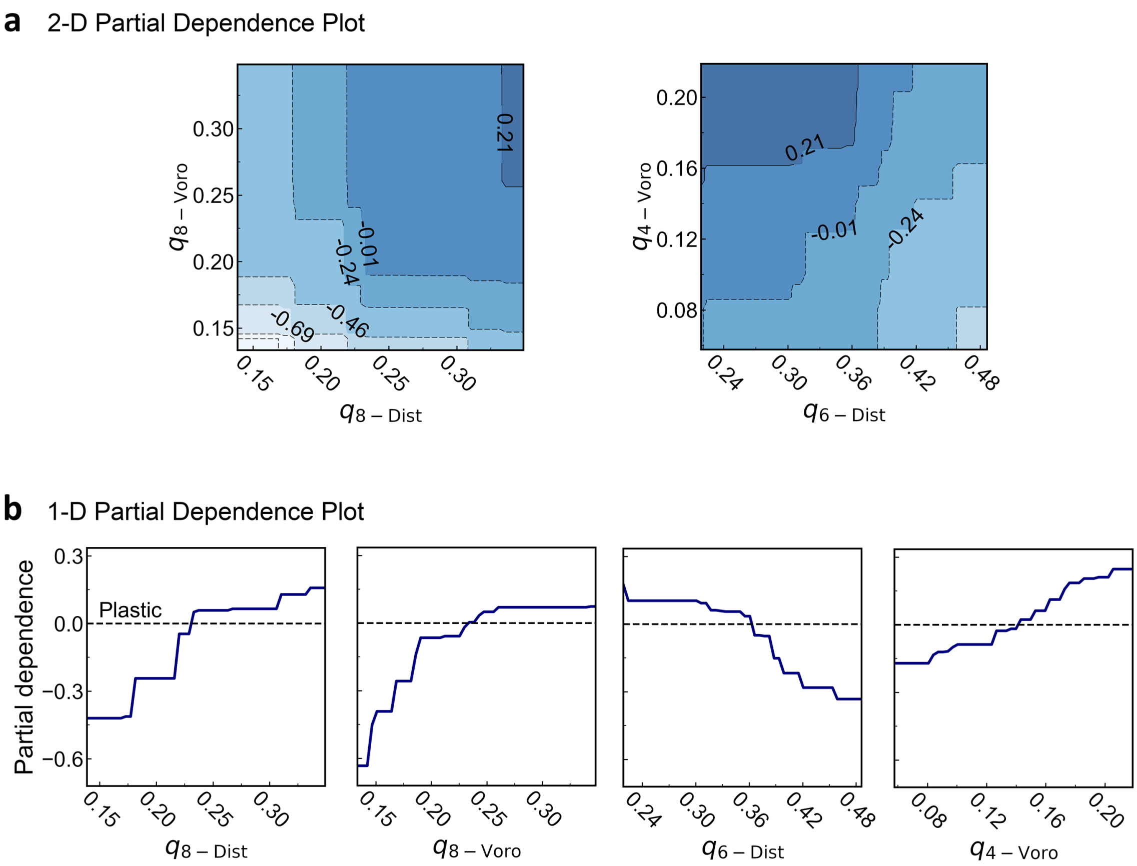

Supplementary Figure 14 | Interpreting the ML model fitted with BOOP $q_{4\ldots10\text{-Voro/Dist}}$ and $w_{4\ldots10\text{-Voro/Dist}}$. (a) Two-dimensional (2-D) and (b) one-dimensional (1-D) partial dependence plots (PDPs) of top 4 features in the ML model are exhibited.

8) $\mathbf{CSRO_{Voro/Dist}}$

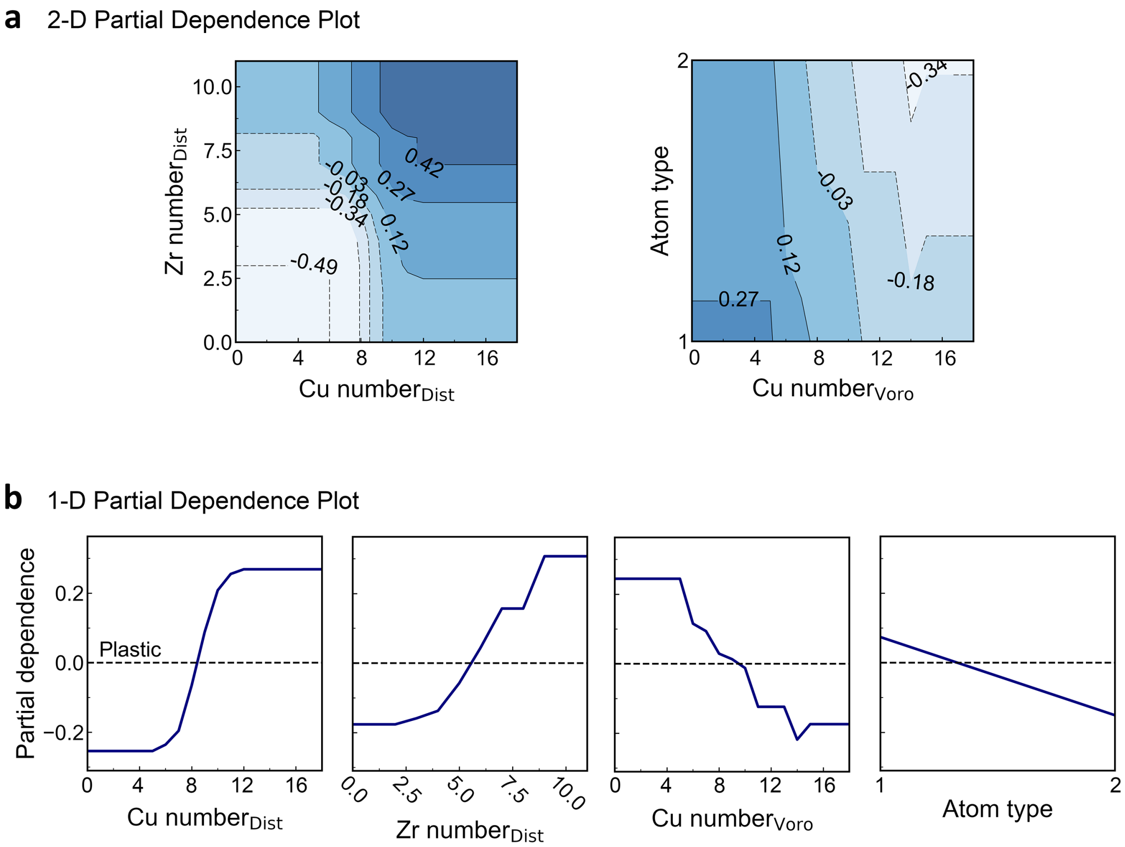

Supplementary Figure 15 | Interpreting the ML model fitted with $CSRO_{Voro/Dist}$. (a) Two-dimensional (2-D) and (b) one-dimensional (1-D) partial dependence plots (PDPs) of top 4 features in the ML model are exhibited.

Medium-range order (MRO) feature sets

1) MRO $CN_{Voro/Dist}$

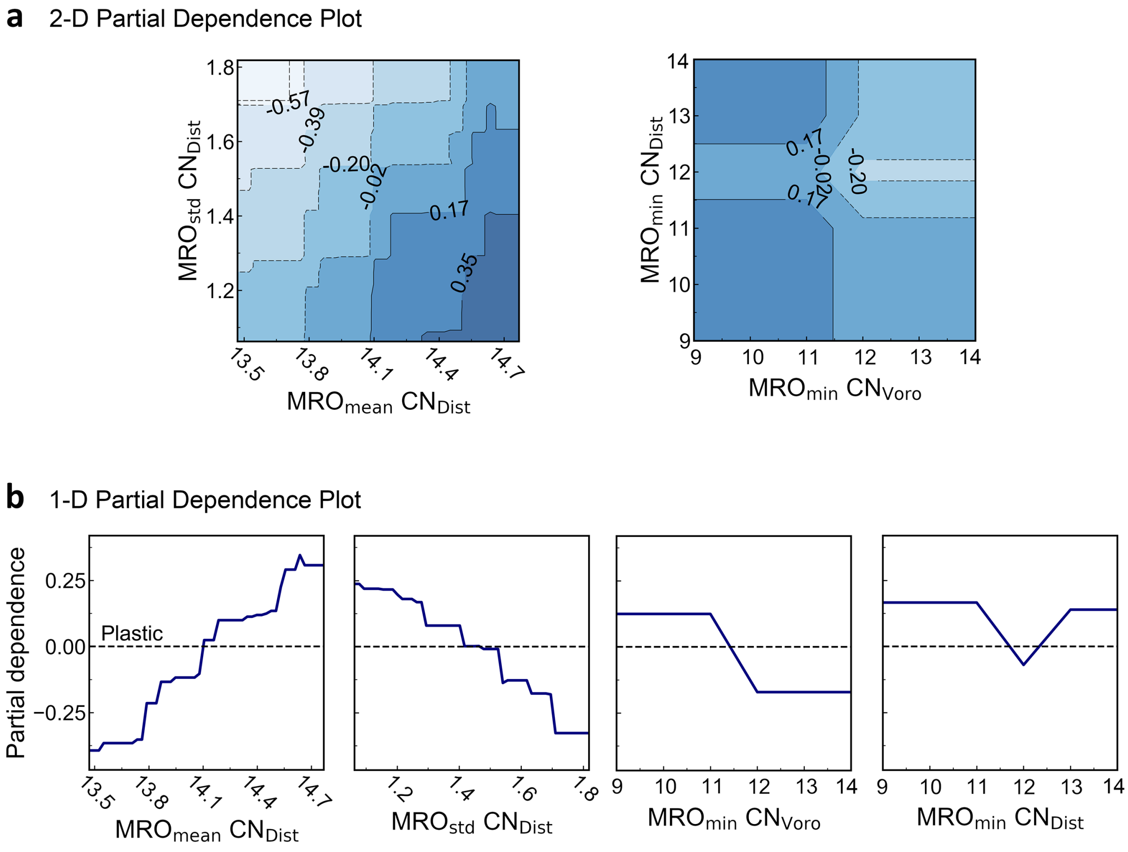

Supplementary Figure 16 | Interpreting the ML model fitted with MRO $CN_{Voro/Dist}$. (a) Two-dimensional (2-D) and (b) one-dimensional (1-D) partial dependence plots (PDPs) of top 4 features in the ML model are exhibited.

2) MRO Voronoi idx$_{3...7}$

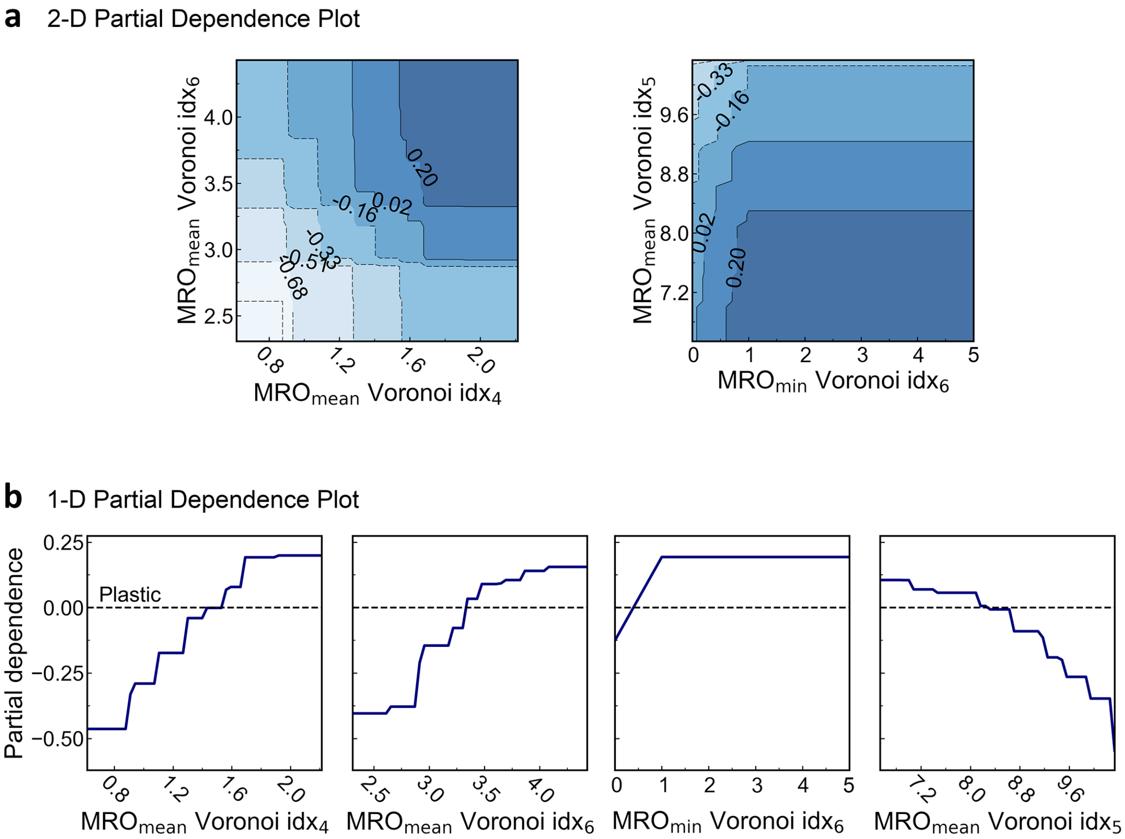

Supplementary Figure 17 | Interpreting the ML model fitted with MRO Voronoi idx$_{3...7}$. (a) Two-dimensional (2-D) and (b) one-dimensional (1-D) partial dependence plots (PDPs) of top 4 features in the ML model are exhibited.

3) MRO Characteristic motifs

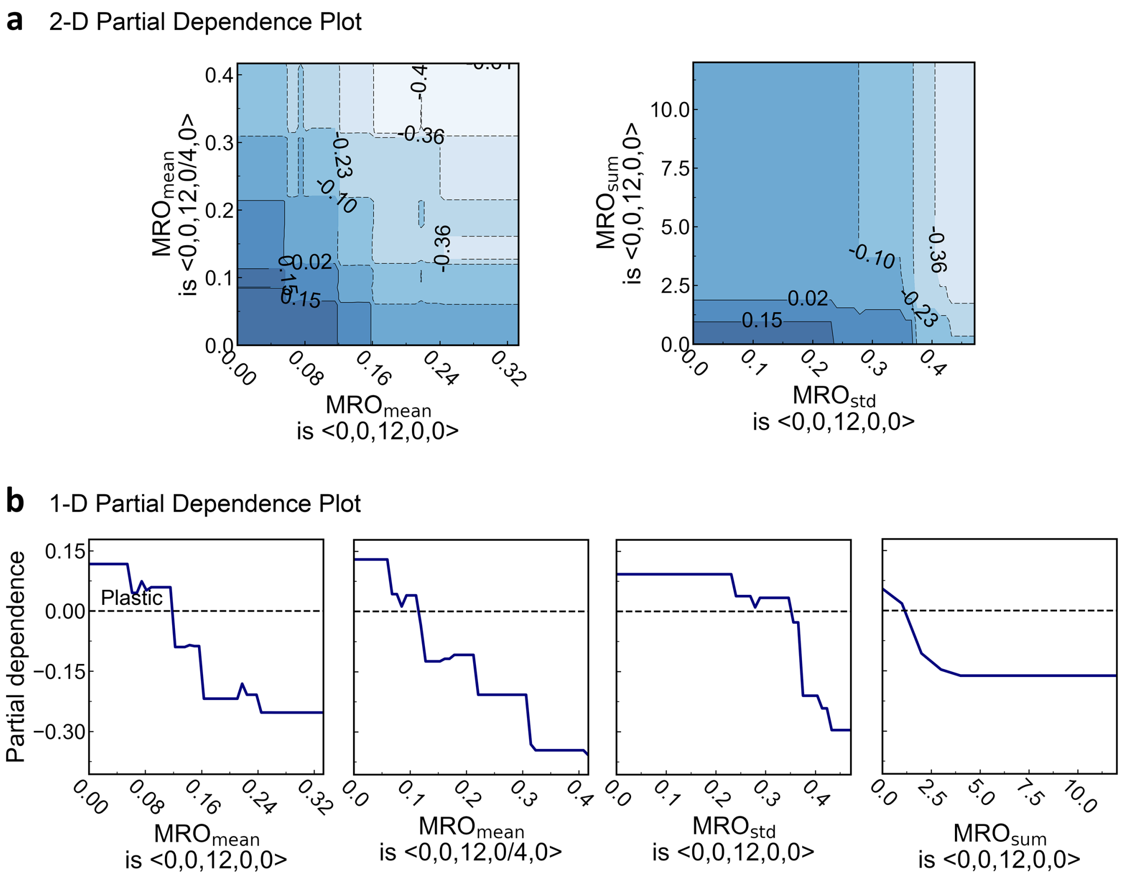

Supplementary Figure 18 | Interpreting the ML model fitted with MRO Characteristic motifs. (a) Two-dimensional (2-D) and (b) one-dimensional (1-D) partial dependence plots (PDPs) of top 4 features in the ML model are exhibited.

4) MRO Volume metrics

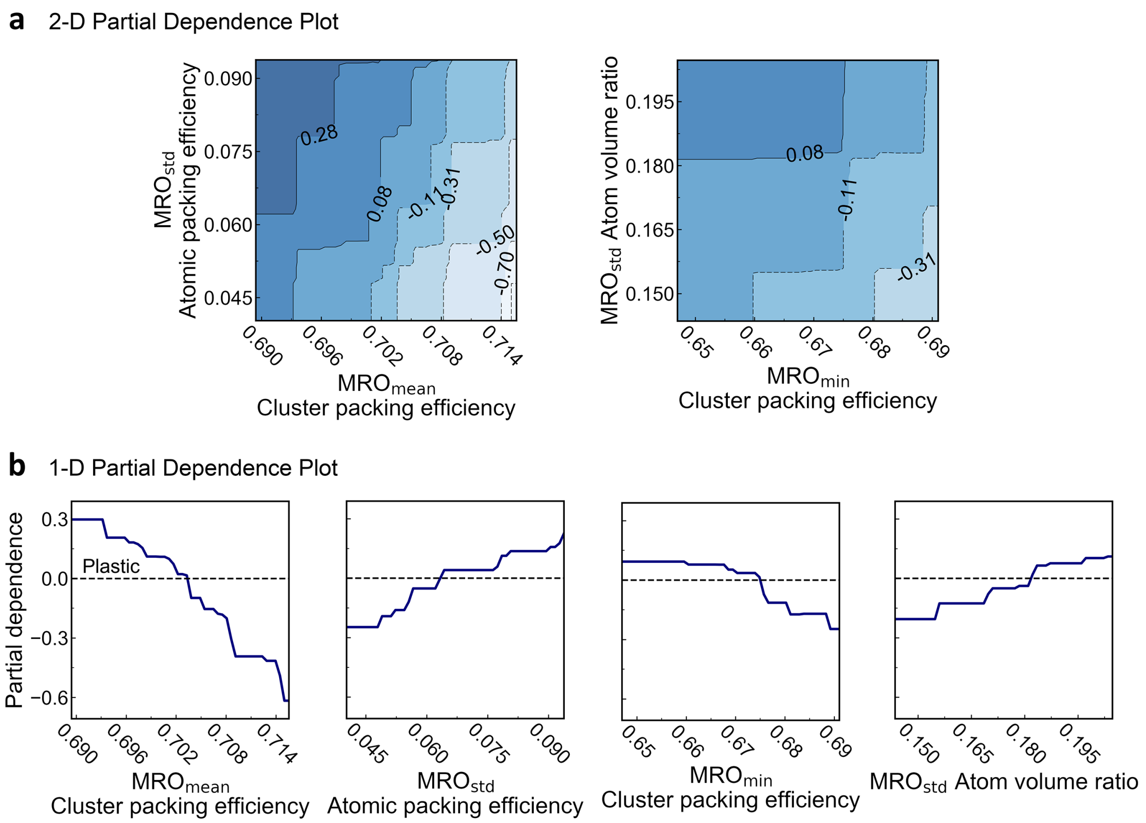

Supplementary Figure 19 | Interpreting the ML model fitted with MRO Volume metrics. (a) Two-dimensional (2-D) and (b) one-dimensional (1-D) partial dependence plots (PDPs) of top 4 features in the ML model are exhibited.

5) Avg. *i*-fold symmetry idx$_{3...7}$

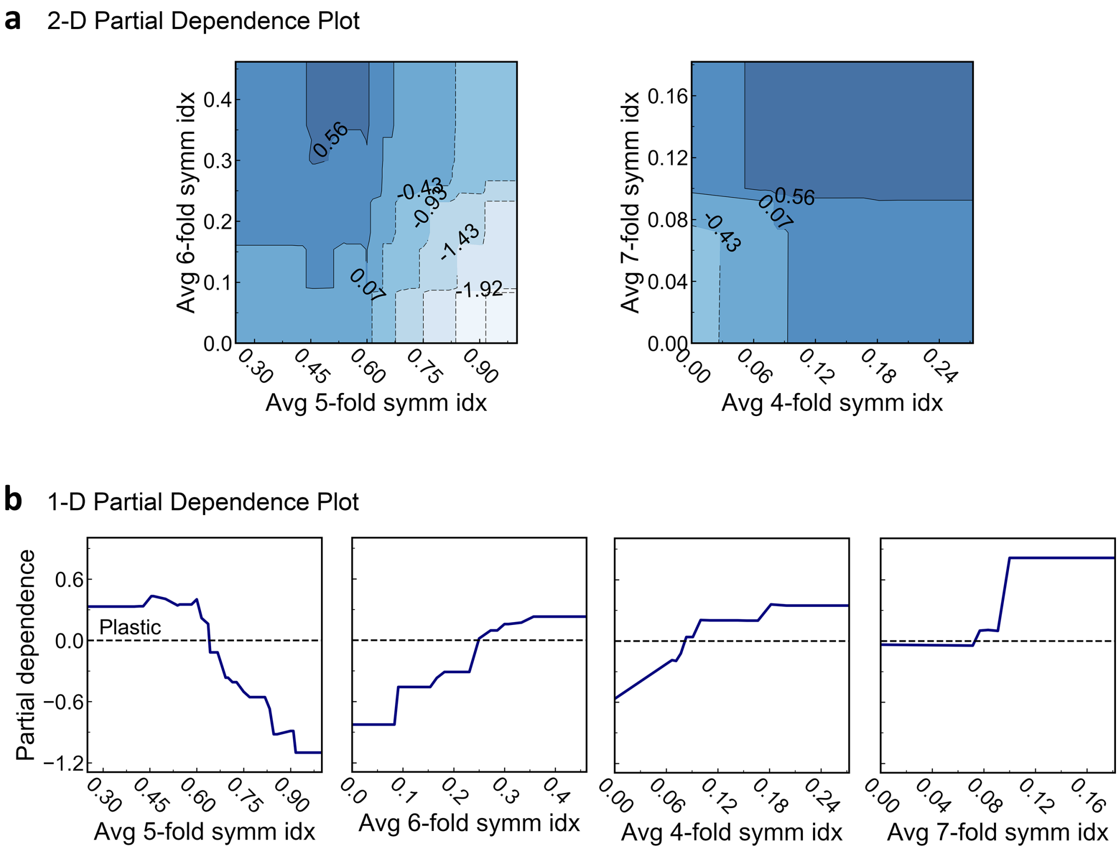

Supplementary Figure 20 | Interpreting the ML model fitted with Avg. *i*-fold symmetry idx$_{3...7}$. (a) Two-dimensional (2-D) and (b) one-dimensional (1-D) partial dependence plots (PDPs) of top 4 features in the ML model are exhibited.

6) MRO *i*-fold symmetry $idx_{3...7}$

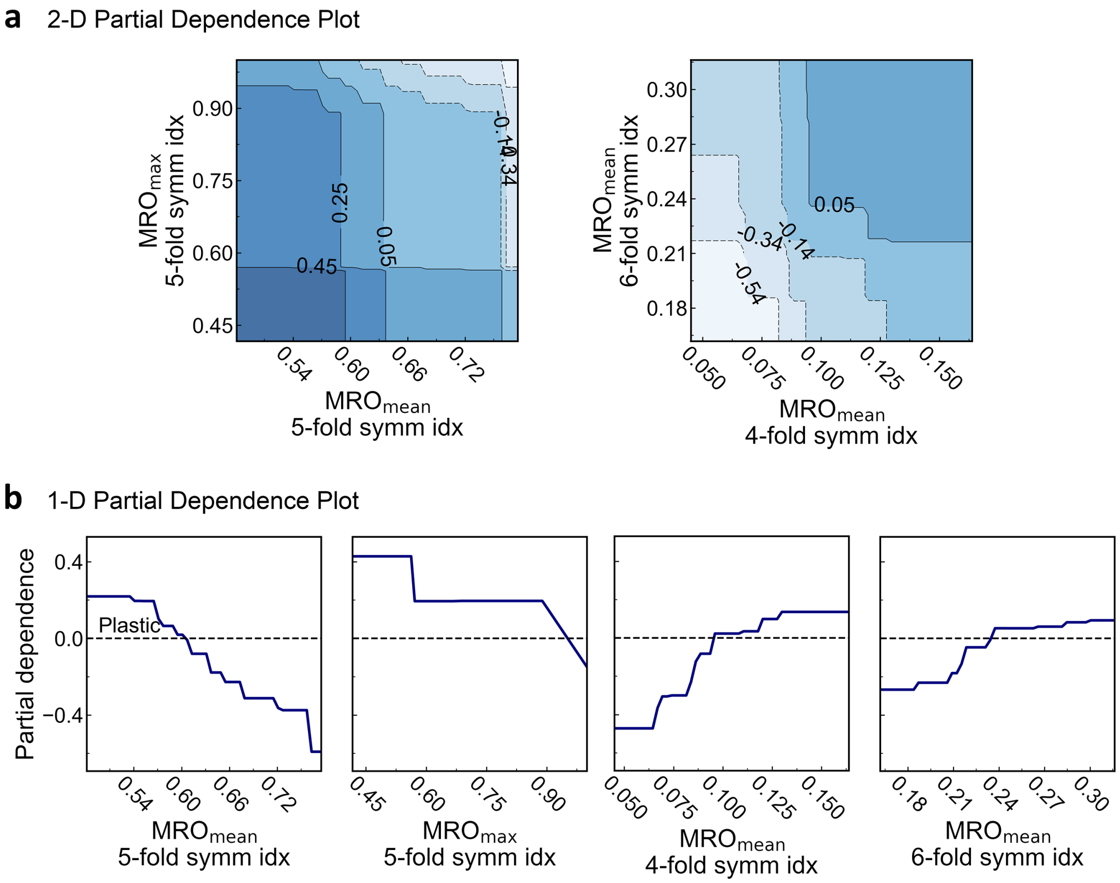

Supplementary Figure 21 | Interpreting the ML model fitted with MRO *i*-fold symmetry $idx_{3...7}$. (a) Two-dimensional (2-D) and (b) one-dimensional (1-D) partial dependence plots (PDPs) of top 4 features in the ML model are exhibited.

7) MRO Weighted *i*-fold symmetry idx$_{3...7}$

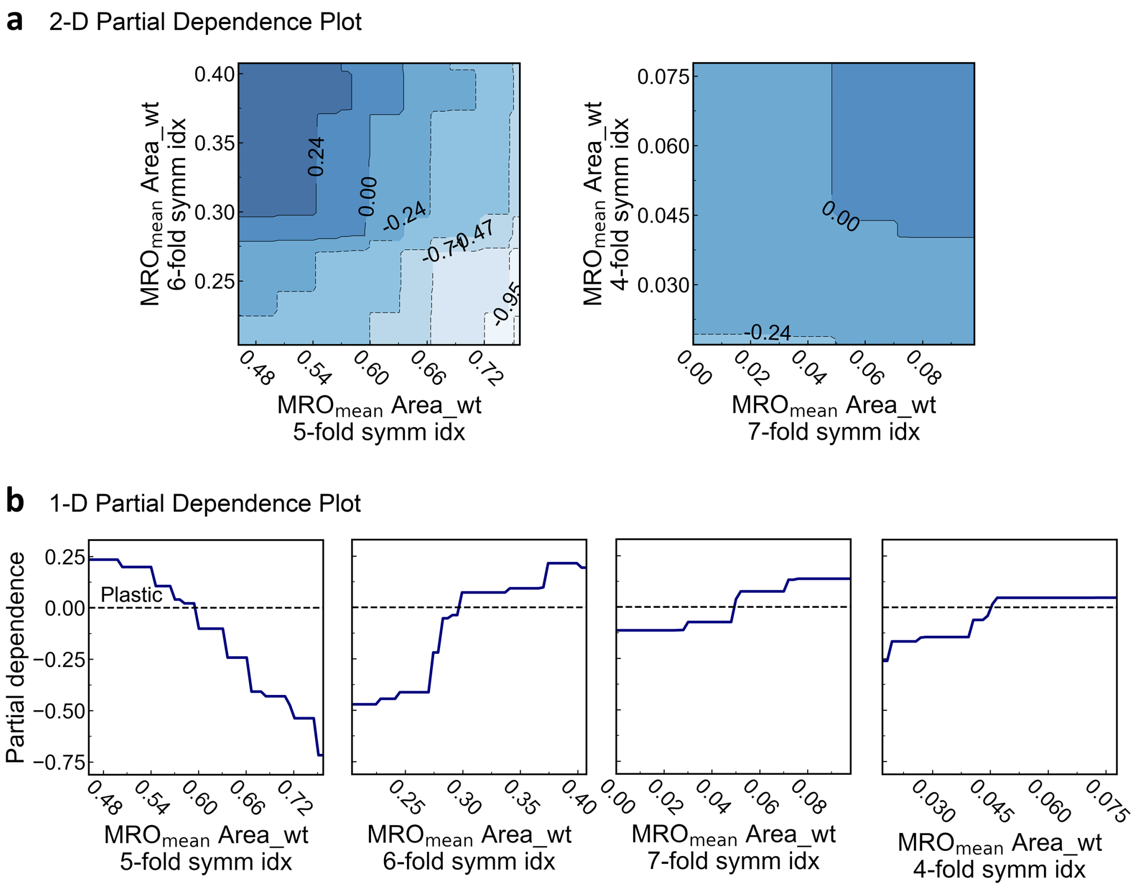

Supplementary Figure 22 | Interpreting the ML model fitted with MRO Weighted *i*-fold symmetry idx$_{3...7}$. (a) Two-dimensional (2-D) and (b) one-dimensional (1-D) partial dependence plots (PDPs) of top 4 features in the ML model are exhibited.

8) Coarse-grained BOOP $q_{4...10\text{-Voro/Dist}}$ and $w_{4...10\text{-Voro/Dist}}$

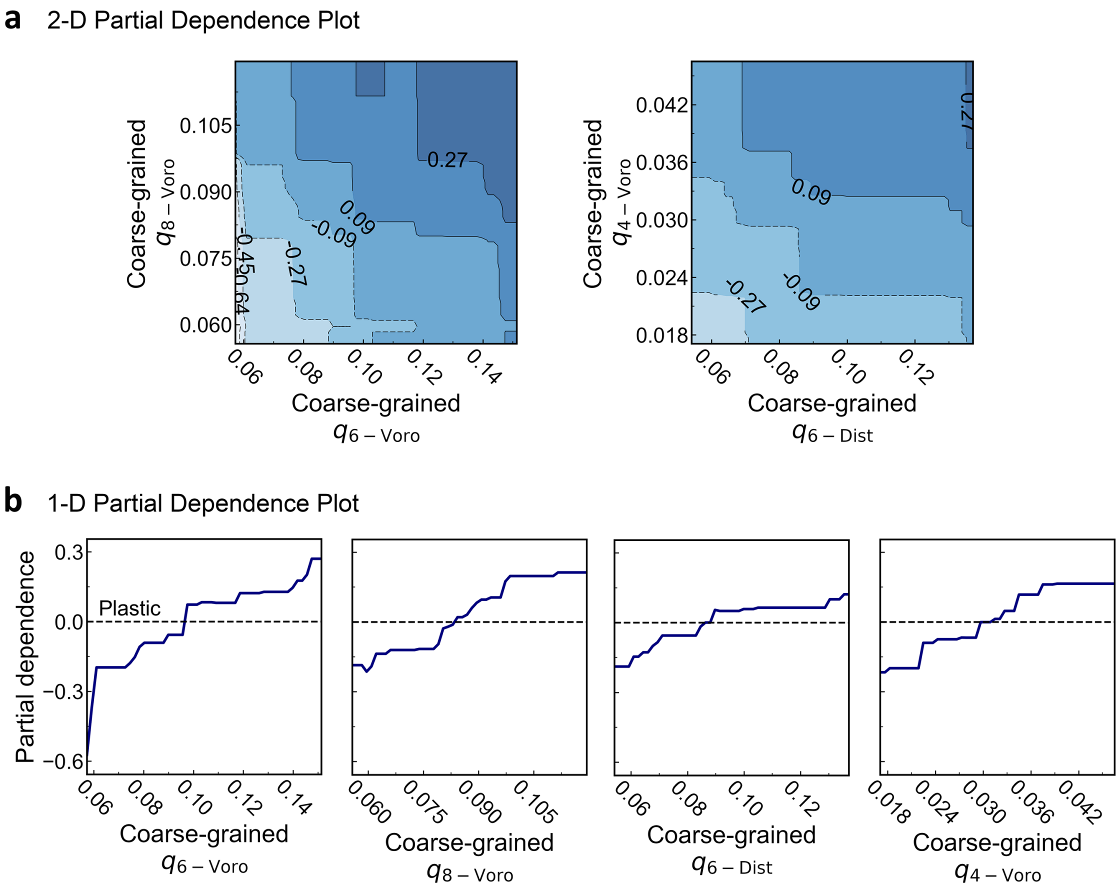

Supplementary Figure 23 | Interpreting the ML model fitted with Coarse-grained BOOP $q_{4...10\text{-Voro/Dist}}$ and $w_{4...10\text{-Voro/Dist}}$. (a) Two-dimensional (2-D) and (b) one-dimensional (1-D) partial dependence plots (PDPs) of top 4 features in the ML model are exhibited.

9) MRO BOOP $q_{4\ldots10\text{-Voro/Dist}}$ and $w_{4\ldots10\text{-Voro/Dist}}$

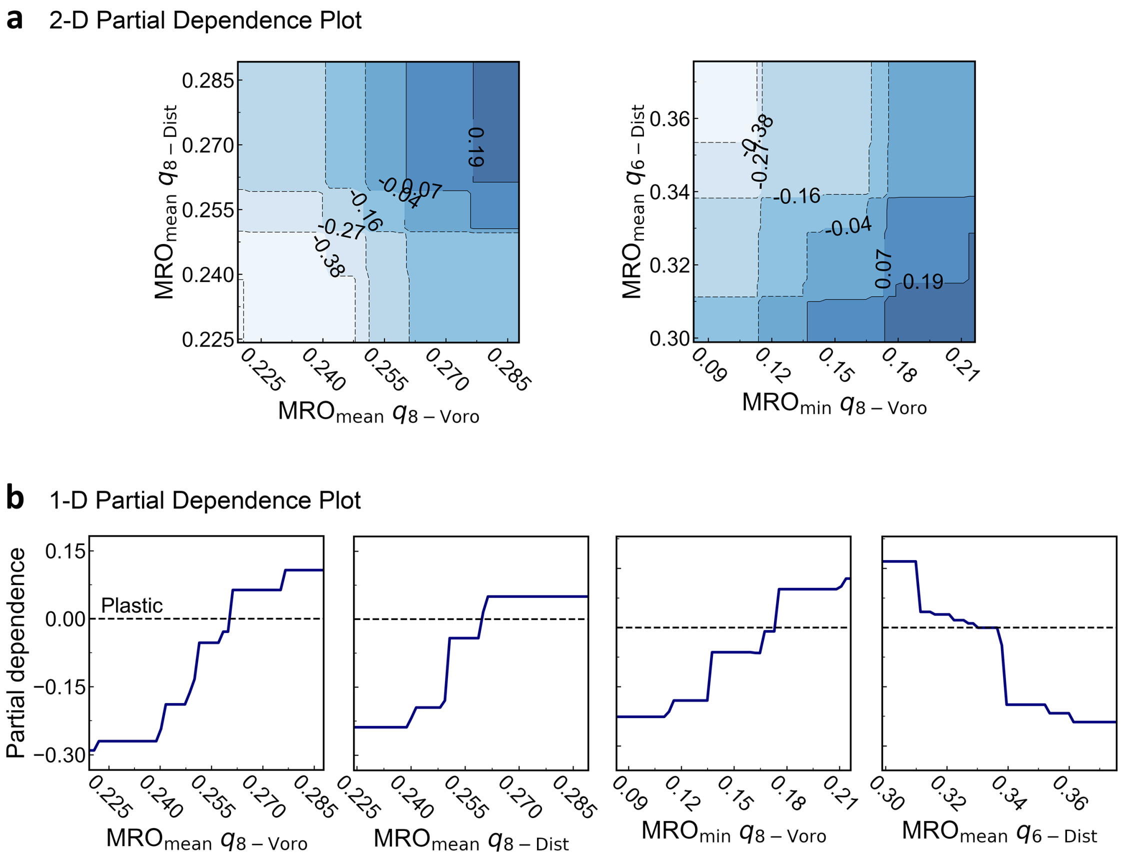

Supplementary Figure 24 | Interpreting the ML model fitted with MRO BOOP $q_{4\ldots10\text{-Voro/Dist}}$ and $w_{4\ldots10\text{-Voro/Dist}}$. (a) Two-dimensional (2-D) and (b) one-dimensional (1-D) partial dependence plots (PDPs) of top 4 features in the ML model are exhibited.

10) $CMRO_{Voro/Dist}$

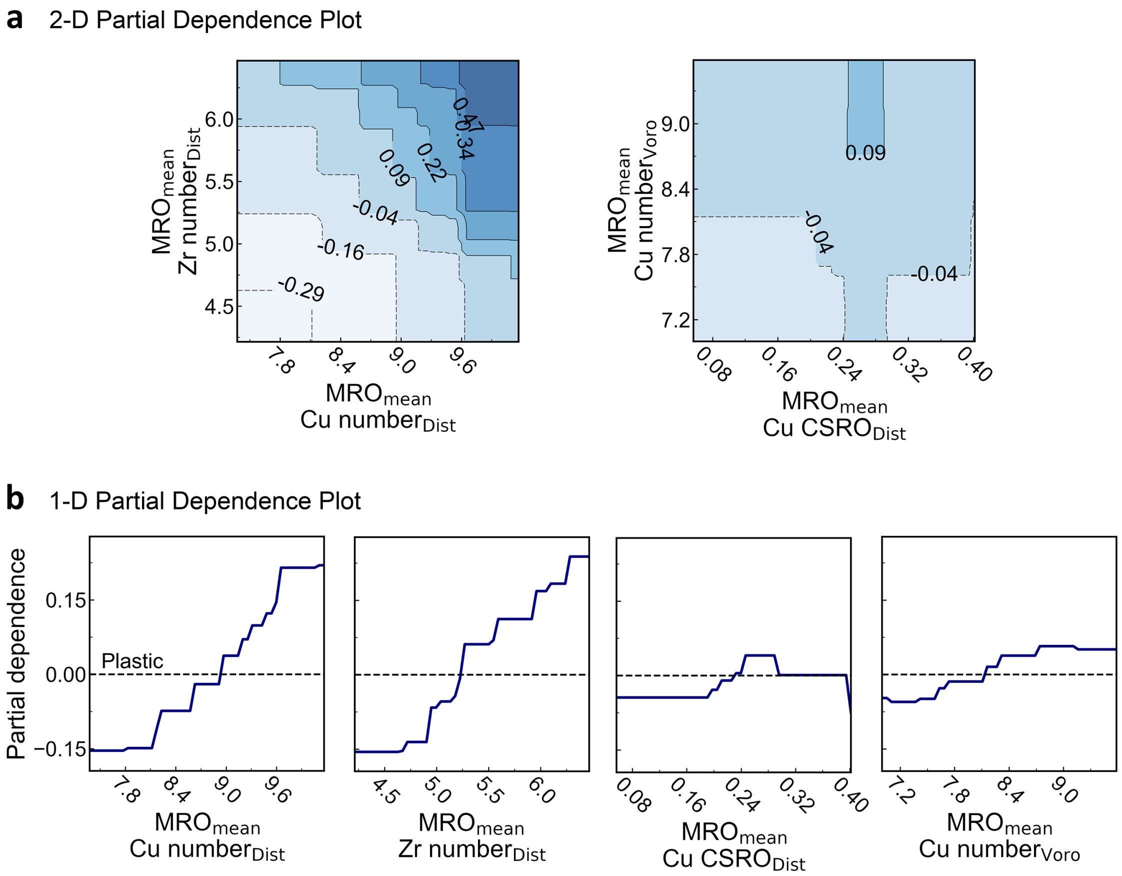

Supplementary Figure 25 | Interpreting the ML model fitted with $CMRO_{Voro/Dist}$. (a) Two-dimensional (2-D) and (b) one-dimensional (1-D) partial dependence plots (PDPs) of top 4 features in the ML model are exhibited.

Supplementary References

1. Cubuk, E. D. *et al.* Identifying structural flow defects in disordered solids using machine-learning methods. *Phys. Rev. Lett.* **114**, 108001 (2015).
2. Cubuk, E. D. *et al.* Structure-property relationships from universal signatures of plasticity in disordered solids. *Science (80-.).* **358**, 1033–1037 (2017).
3. Schoenholz, S. S., Cubuk, E. D., Sussman, D. M., Kaxiras, E. & Liu, A. J. A structural approach to relaxation in glassy liquids. *Nat. Phys.* **12**, 469–471 (2016).
4. Falk, M. L. & Langer, J. S. Dynamics of viscoplastic deformation in amorphous solids. *Phys. Rev. E - Stat. Physics, Plasmas, Fluids, Relat. Interdiscip. Top.* **57**, 7192–7205 (1998).
5. Stukowski, A. Visualization and analysis of atomistic simulation data with OVITO-the Open Visualization Tool. *Model. Simul. Mater. Sci. Eng.* **18**, (2010).
6. Behler, J. & Parrinello, M. Generalized Neural-Network Representation of High-Dimensional Potential-Energy Surfaces. *Phys. Rev. Lett.* **98**, 146401 (2007).
7. Bartók, A. P., Kondor, R. & Csányi, G. On representing chemical environments. *Phys. Rev. B* **87**, 184115 (2013).
8. Okabe, A., Boots, B., Sugihara, K. & Chiu, S. N. *Spatial Tesselations. Concepts and Applications of Voronoi Diagrams* (2009). doi:10.1002/0471721182.scard
9. Frank, F. C. & Kasper, J. S. Complex alloy structures regarded as sphere packings. I. Definitions and basic principles. *Acta Crystallogr.* **11**, 184–190 (1958).
10. Sheng, H. W., Luo, W. K., Alamgir, F. M., Bai, J. M. & Ma, E. Atomic packing and short-to-medium-range order in metallic glasses. *Nature* **439**, 419–425 (2006).
11. Steinhardt, P. J., Nelson, D. R. & Ronchetti, M. Bond-orientational order in liquids and glasses. *Phys. Rev. B* **28**, 784–805 (1983).
12. Yang, L. *et al.* Atomic-Scale Mechanisms of the Glass-Forming Ability in Metallic Glasses. *Phys. Rev. Lett.* **109**, 105502 (2012).
13. Laws, K. J., Miracle, D. B. & Ferry, M. A predictive structural model for bulk metallic glasses. *Nat. Commun.* **6**, 8123 (2015).
14. Peng, H. L., Li, M. Z. & Wang, W. H. Structural signature of plastic deformation in metallic glasses. *Phys. Rev. Lett.* **106**, 135503 (2011).
15. Cowley, J. M. An approximate theory of order in alloys. *Phys. Rev.* **77**, 669–675 (1950).
16. Warren, B. E. X-ray diffraction. *Analysis* **1**, 402 (1990).
17. Lechner, W. & Dellago, C. Accurate determination of crystal structures based on averaged local bond order parameters. *J. Chem. Phys.* **129**, 114707 (2008).
18. https://scikit-learn.org/stable/modules/calibration.html.

19. Platt, J. Probabilistic outputs for support vector machines and comparisons to regularized likelihood methods. *Adv. large margin Classif.* **10**, 61–74 (1999).

20. Barlow, R. E., Bartholomew, D. J., Bremner, J. M. & Brunk, H. D. *Statistical inference under order restrictions: The theory and application of isotonic regression.* (Wiley New York, 1972).
